# Epicardial placement of human placental membrane protects from heart injury in a swine model of myocardial infarction

**DOI:** 10.14814/phy2.15838

**Published:** 2023-10-17

**Authors:** Rinku S. Skaria, Marissa A. Lopez‐Pier, Brij S. Kathuria, Christian J. Leber, Paul R. Langlais, Shravan G. Aras, Zain I. Khalpey, Pamela G. Hitscherich, Evangelia Chnari, Marc Long, Jared M. Churko, Raymond B. Runyan, John P. Konhilas

**Affiliations:** ^1^ Department of Physiology University of Arizona College of Medicine Tucson Arizona USA; ^2^ Department of Biomedical Engineering University of Arizona College of Engineering Tucson Arizona USA; ^3^ Department of Medicine University of Arizona College of Medicine Tucson Arizona USA; ^4^ Center for Biomedical and Informatics University of Arizona Health Sciences Tucson Arizona USA; ^5^ Northwest Healthcare Tucson Arizona USA; ^6^ MTF Biologics Edison New Jersey USA; ^7^ Department of Cellular and Molecular Medicine University of Arizona College of Medicine Tucson Arizona USA; ^8^ Sarver Molecular Cardiovascular Research Program University of Arizona College of Medicine Tucson Arizona USA

## Abstract

Cardiac ischemic reperfusion injury (IRI) is paradoxically instigated by reestablishing blood‐flow to ischemic myocardium typically from a myocardial infarction (MI). Although revascularization following MI remains the standard of care, effective strategies remain limited to prevent or attenuate IRI. We hypothesized that epicardial placement of human placental amnion/chorion (HPAC) grafts will protect against IRI. Using a clinically relevant model of IRI, swine were subjected to 45 min percutaneous ischemia followed with (MI + HPAC, *n* = 3) or without (MI only, *n* = 3) HPAC. Cardiac function was assessed by echocardiography, and regional punch biopsies were collected 14 days post‐operatively. A deep phenotyping approach was implemented by using histological interrogation and incorporating global proteomics and transcriptomics in nonischemic, ischemic, and border zone biopsies. Our results established HPAC limited the extent of cardiac injury by 50% (11.0 ± 2.0% vs. 22.0 ± 3.0%, *p* = 0.039) and preserved ejection fraction in HPAC‐treated swine (46.8 ± 2.7% vs. 35.8 ± 4.5%, *p* = 0.014). We present comprehensive transcriptome and proteome profiles of infarct (IZ), border (BZ), and remote (RZ) zone punch biopsies from swine myocardium during the proliferative cardiac repair phase 14 days post‐MI. Both HPAC‐treated and untreated tissues showed regional dynamic responses, whereas only HPAC‐treated IZ revealed active immune and extracellular matrix remodeling. Decreased endoplasmic reticulum (ER)‐dependent protein secretion and increased antiapoptotic and anti‐inflammatory responses were measured in HPAC‐treated biopsies. We provide quantitative evidence HPAC reduced cardiac injury from MI in a preclinical swine model, establishing a potential new therapeutic strategy for IRI. Minimizing the impact of MI remains a central clinical challenge. We present a new strategy to attenuate post‐MI cardiac injury using HPAC in a swine model of IRI. Placement of HPAC membrane on the heart following MI minimizes ischemic damage, preserves cardiac function, and promotes anti‐inflammatory signaling pathways.

## 1 INTRODUCTION

Myocardial infarction (MI) secondary to coronary artery disease (CAD) remains the most common cause of heart failure (HF), costing over $30 billion in healthcare costs. (Alkhouli et al., 2020; Tsao et al., 2023) Early revascularization after an MI by coronary bypass graft (CABG) surgery or percutaneous coronary intervention (PCI; angioplasty with stent) is the most effective strategy to salvage myocardium, reduce infarct size, and prevent HF. (Hausenloy & Yellon, 2013; Neri et al., 2017; O'Neill et al., 1986) However, restoration of blood flow to the oxygen‐deprived heart generates energetic imbalances, oxidative stress, and a massive inflammatory response that further damages the heart, (Cardilo‐Reis et al., 2010) a paradox termed “myocardial ischemia and reperfusion injury” (IRI). (Nahrendorf et al., 2007; Panizzi et al., 2010) These biological cues dictate the size of the initial infarct and quality of infarct healing, key elements that determine long‐term survival and progression to HF. (Gaudron et al., 1993; White et al., 1987) Therefore, delaying pro‐inflammatory while promoting anti‐inflammatory mediators early on, produces long‐term benefits and better clinical outcomes. (Matsumoto et al., 2011; Tang et al., 2012) Despite advances in revascularization procedures, no effective therapeutic strategy for preventing myocardial IRI injury exists, resulting in a major clinical gap.

Human placental tissue is a designated 361 human cell and tissue‐based product and has been shown to be safe as a wound covering for internal and external wound use. Human placental tissue without donor‐host tissue matching has been used since 1910 and does not cause acute rejection. (Adinolfi, 1982; Adinolfi et al., 1982; Akle et al., 1981; Delo et al., 2006) Human placental tissue is non‐immunogenic and has been used safely in a variety of clinical applications to treat wounds, burns, diabetic and chronic leg ulcers. (Colocho et al., 1974; DiDomenico et al., 2018; Prasad et al., 1986; Serena et al., 2022; Subrahmanyam, 1995; Trelford & Trelford‐Sauder, 1979) Our group recently established proof‐of‐concept human placental graft protocols and use for cardiac surgical applications. (Khalpey et al., 2015) Processing of donated placental tissues yields a multipotent tissue matrix containing endogenous cytokines, proteins, growth factors, extracellular matrix, fibronectin, and laminin, associated with anti‐inflammatory, angiogenic, antibacterial, and anti‐fibrotic properties. (Chau et al., 2012; Diaz‐Prado et al., 2010; Niknejad et al., 2008; Ricci et al., 2013; Tang et al., 2010; Wen et al., 2013)

Accordingly, our hypothesis states, placement of human placental amnion/chorion (HPAC) grafts as a wound covering over the heart tissue attenuates IRI injury. We previously observed promising long‐term cardiac remodeling with preserved function and morphometrics in a murine model (Figures S5 and S6). When placed on the surface of mouse or pig hearts following an ischemic insult, we found HPAC membranes preserved cardiac function and reduced infarct size, indicators of delayed progression to HF. (Hausenloy & Yellon, 2013; Neri et al., 2017; O'Neill et al., 1986) Assuming positive results in an animal model in this study, advancement to clinical use will be contingent on a clear in vivo enhancement of long‐term cardiac function post‐MI coupled with a robust, deep phenotyping of the cellular and molecular mechanisms underlying HPAC efficacy. Therefore, we also employed transcriptomic and proteomic analysis of (Tsao et al., 2023) ischemic tissue (within scar/necrotic area), (Alkhouli et al., 2020) border zone (interface between ischemic and nonischemic tissue), and (Hausenloy & Yellon, 2013) nonischemic tissue (outside ischemic area and border zone).

## 2 MATERIALS AND METHODS

Swine Model: An expanded version of Methods is provided in the supplemental information. All experiments were performed using protocols that adhered to guidelines and approved by the Institutional Animal Care and Use Committee at the University of Arizona and to 2019 NIH guidelines for care and use of laboratory animals. We utilized an MI swine model and procedure that abides by the NHLBI‐sponsored Consortium for preclinical assESsment of cARdioprotective therapies (CAESAR) and American Heart Association. Swine is the optimal candidate and lowest animal species that has equivalent genetics, physiology, and structural features comparable to humans. Importantly, the swine model of MI is recognized as a valid, relevant preclinical model to predict clinical outcomes.

A short‐term (14‐day) study, post‐MI, was performed on swine to examine responses to HPAC in various regions of the heart. A total of eight 3–4 month‐old male domestic farm pigs, weighing 26–43 kg, were initially included in the study and randomly assigned to groups receiving a MI only and MI plus an aseptically processed, amnion and chorion membranes (MI + HPAC; AmnioBand® Membrane, MTF Biologics®, Edison, NJ) from donated human placentas). (Dolivo et al., 2021) Four pigs served as controls (no MI, no HPAC). The survival rate was 92%, resulting in a final number of 11 animals completing the protocol. Figure S1 shows the experimental design. Following the end of the 45‐minute occlusion ischemic period, swine in the MI + HPAC group underwent a median hemi‐sternotomy. The LV was exposed, and the HPAC graft (5cm x 5cm) was sutured to the infarcted zone using 6–0 prolene.

### 2.1 Echocardiography

Transthoracic echocardiography was performed using a GE LOGIQ E imaging system (GE Medical Systems, USA) to noninvasively examine morphological and functional changes associated with HPAC application. Swine in MI only and MI + HPAC groups underwent echocardiography pre‐MI, post‐MI, and postoperative day 14 (POD 14).

### 2.2 Tissue harvest and preservation

After 14 days following surgical procedure, the pig heart was excised within 5 min and immediately placed on ice (Figure S2). Cardiac tissue was cored using a 10 mm punch biopsy from three distinct zones of the ischemic heart: (1) infarct zone (IZ; scar/necrotic tissue), (2) border zone (BZ; interface between ischemic area and viable tissue), and (3) remote zone (RZ; outside infarct and border zone). Three samples from each zone were obtained for RNA, proteome, and histology analyses.

### 2.3 Determination of infarct size and histological staining

Upon extraction of the pig LV cores, the explanted heart was semi‐frozen at −20°C and sectioned from apex to base into 0.5 cm thick short‐axis segments using a meat slicer. The slices were weighed and then stained with 1% 2,3,4‐triphenyl tetrazolium chloride (TTC), (Sigma‐Aldrich) solution to distinguish live and dead tissue. Slices were digitally photographed at 8X magnification and measured for each section using image analysis software (Image J 1.52a). Infarction size was reported as a percentage of area of necrosis (AON) to left ventricle (LV). Six assessors were blinded and independently evaluated the slices.

### 2.4 RNA sequencing and proteomics

RNA sequencing was performed by Novogene (Sacramento, CA) using Illumina platform (Illumina, San Diego, CA), and global proteomics of punch biopsies executed by mass spectrometry, respectively, followed by quality assurance and data analysis. A detailed description of the extraction and analysis are provided in the Supplemental Methods. The data discussed in this publication have been deposited in NCBI's Gene Expression Omnibus (Edgar et al., 2002) and are accessible through GEO Series accession number GSE228096.

### 2.5 Western blotting

All immunoblot analysis (Table S1) was performed from the semi‐quantitation of individual blots and was not compared across blots according to accepted guidelines. Immunoblot images of a given target were cropped from the same blot in order to conserve figure space and redundancy (see supplemental materials for detailed methods).

### 2.6 Statistical analysis

Results are presented as mean ± SEM, unless otherwise stated. Statistical analysis was performed using commercially available software (GraphPad Prism 8.3.1, GraphPad, La Jolla, CA). Comparisons between two groups were made using paired or unpaired *t*‐test, where appropriate. Regional differences in protein expression (Western Blotting) among experimental groups were analyzed by one‐way ANOVA followed by a Tukey's HSD post hoc test. For all statistical analyses, significance was accepted at *p* < 0.05. Supplemental data is provided through the following link https://figshare.com/s/6004af0b05f1cca5b935.

## 3 RESULTS

### 3.1 HPAC preserved cardiac function and structure after MI in a swine model

**FIGURE 1:**
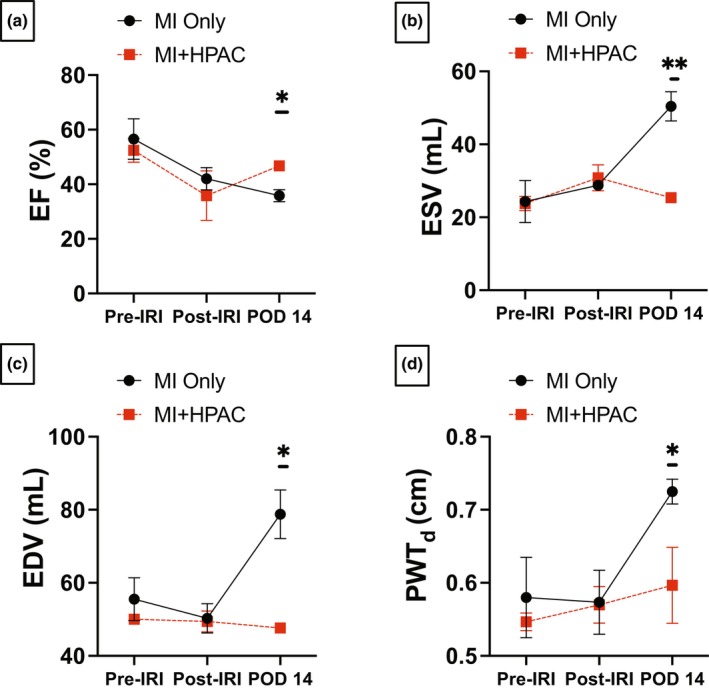
HPAC membrane preserved cardiac function. Echocardiographic parameters were measured Pre‐IRI and Post‐IRI and on postoperative day 14 (POD 14) in MI only (black circle; *n* = 3) and MI + HPAC (red square; *n* = 3) groups. (a) EF decreased in both groups post‐IRI with nearly restored EF in MI + HPAC group by POD 14 (*p = 0.014). (b) Preserved ESV (**p = 0.0036) and (c) EDV (*p = 0.011) are noted in MI + HPAC group by POD 14. (d) PWT_d_ was significantly increased (*p = 0.0442) in MI only group. EDV, end diastolic volume; EF, ejection fraction; ESV, end systolic volume; PWT_d_, posterior wall thickness. Data is presented as mean ± S.E.M.

To evaluate the impact and underlying mechanisms of HPAC tissue in a non‐rodent and more physiologically relevant preclinical model, we utilized a swine model of IRI. The pig model of IRI exhibits similar hemodynamics and is recognized as a validated, translationally relevant preclinical model to predict clinical outcomes. (Cibelli et al., 2013; Harding et al., 2013; van der Spoel et al., 2011) Three groups of swine (*n* = 3 per group) were used in this study: control, MI only, and MI + HPAC. Echocardiographic parameters are summarized in Table 1 and graphically displayed in bar graph form (Figure 1). As expected, ejection fraction (EF) at baseline (Pre‐IRI) decreased immediately following the IRI protocol (Post‐IRI) in both experimental groups (Figure 1a). Prior to sacrifice on POD 14, EF continued to decline, although not significantly, in MI only pigs, whereas EF was significantly (*p* = 0.014) improved in swine receiving HPAC over MI only. In situ echocardiography also demonstrated that HPAC placement significantly protected ventricular morphometry measured at POD 14 as evidenced by maintained end systolic volume (ESV) (*p* = 0.0036) and end diastolic volume (EDV) (*p* = 0.011) (Figure 1b, c). Finally, HPAC placement protected against regional cardiac hypertrophy due to MI demonstrated by a significant (*p* = 0.0442) attenuation of posterior wall thickness (PWT) in the MI + HPAC group compared to MI only group (Figure 1d). Otherwise, there were no significant findings across groups or timepoints for heart rate (HR), stroke volume (SV), cardiac output (CO), Left Ventricular endocardial Length (LVL), and Doppler measurements.

**TABLE 1:** Echocardiographic Parameters of MI Only and MI + HPAC‐Treated Hearts.

		MI only (*n* = 3)		MI + HPAC (*n* = 3)		
Parameter	Time	AVG	SEM	AVG	SEM	*p*‐Value
EF (%)	Pre‐IRI	56.5	7.4	52.4	4.3	ns
	Post‐IRI	42.1	4.1	35.8	9.1	ns
	POD 14	34.0	1.9	46.8	1.6	*p = 0.014
ESV (mL)	Pre‐IRI	24.4	5.8	23.8	2.0	ns
	Post‐IRI	28.8	0.7	30.8	3.5	ns
	POD 14	45.9	5.0	25.4	1.2	*p = 0.0036
EDV (mL)	Pre‐IRI	55.5	5.8	50.0	1.1	ns
	Post‐IRI	50.3	4.0	49.4	2.8	ns
	POD 14	73.4	5.6	47.6	0.8	*p = 0.011
PWTd (cm)	Pre‐IRI	0.58	0.06	0.55	0.01	ns
	Post‐IRI	0.57	0.04	0.57	0.03	ns
	POD 14	0.71	0.01	0.60	0.05	*p = 0.044

*Note*: Values are presented as Mean ± S.E.M.

Abbreviations: EDV, end diastolic volume; EF, ejection fraction; ESV, end systolic volume; Pre‐IRI, prior to infarct; Post‐IRI, following infarct; POD 14, postoperative day 14 (prior to sacrifice); PWTd, posterior wall thickness in end diastole.

### 3.2 HPAC attenuated adverse myocardial tissue remodeling after MI

**FIGURE 2:**
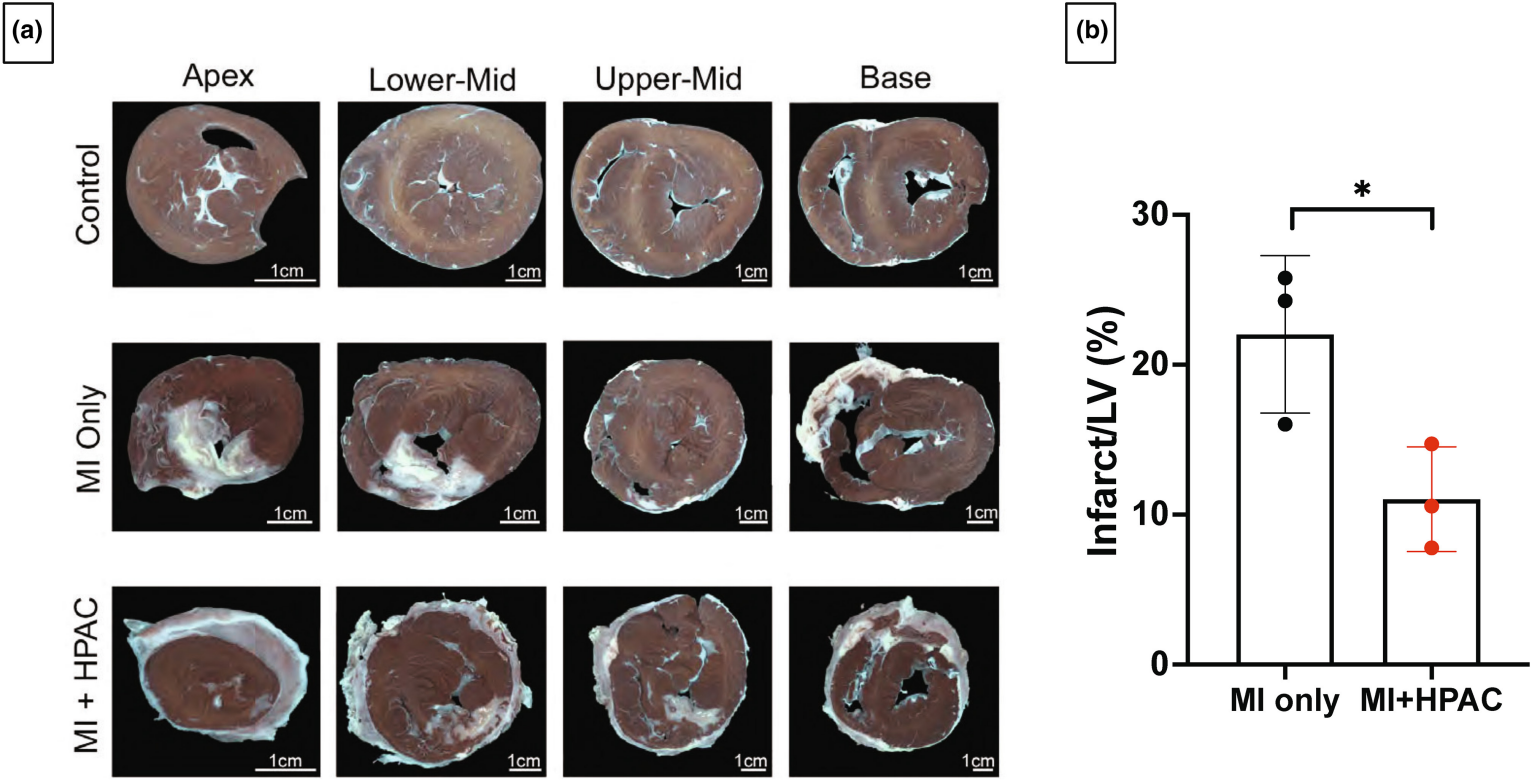
Infarct size was attenuated by HPAC membrane. After sacrifice, hearts were excised, and 0.5 cm thick short‐axis segments were stained with 1% 2,3,4‐triphenyl tetrazolium chloride (TTC) to determine infarct size. (a) Representative images of transverse slices between cardiac apex and base from the three groups (*n* = 3 per group) are shown with viable myocardium stained red and infarcted tissue remaining white. Scale bars = 1 cm. (b) Quantitative analysis demonstrated significant (*p = 0.039) decrease in infarct size to left ventricle (LV) area with HPAC membrane application. Data is presented as mean ± S.E.M.

**FIGURE 3:**
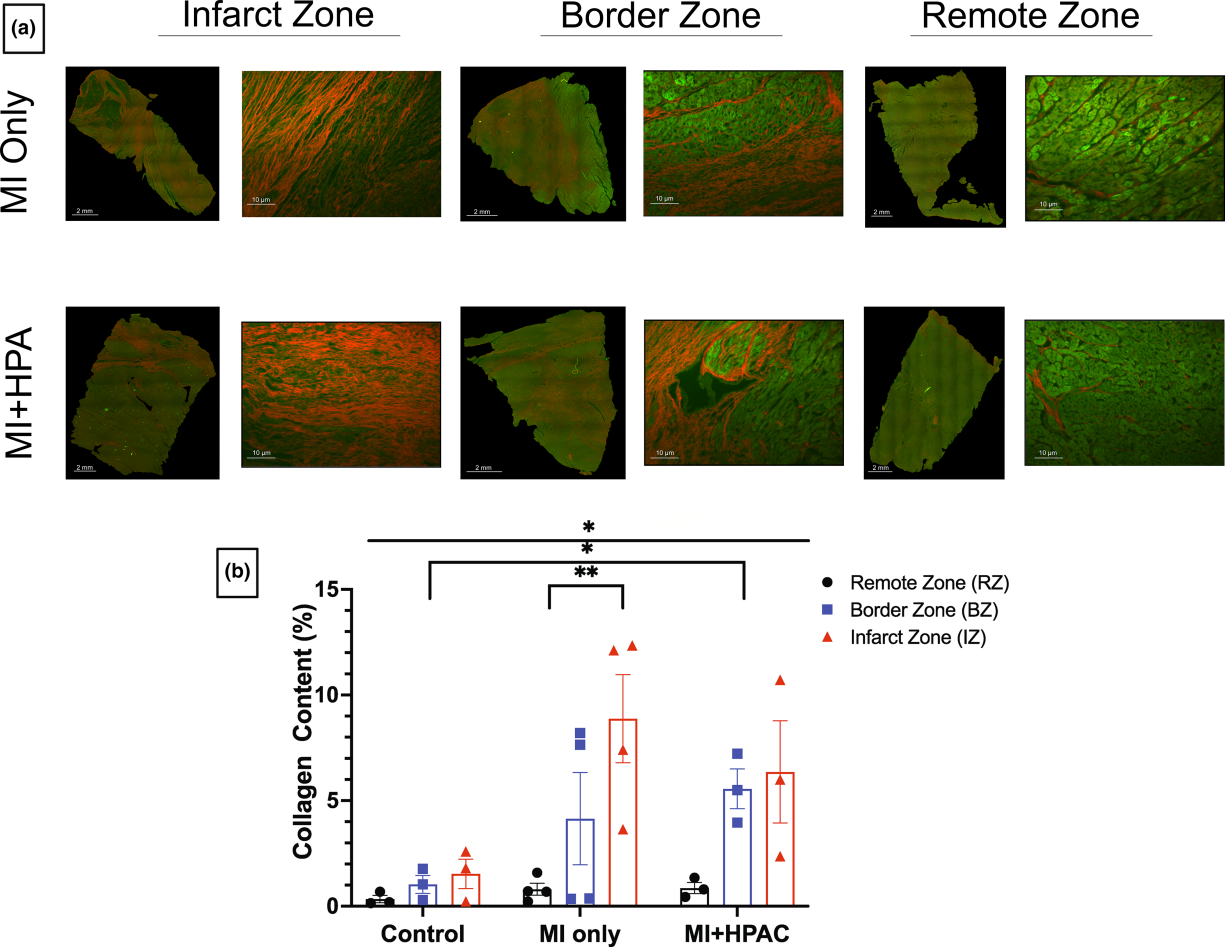
Cardiac remodeling was evident in swine infarct and border zone. Punch biopsies from remote (nonischemic), border, and infarct zone were excised, fixed, and stained with picrosirius red (PSR) 14 days post ischemia reperfusion injury. Fibrosis is visualized as red fluorescence in PSR‐stained hearts using a Texas red fluorescence filter, and viable, non‐fibrotic tissue is visualized as green. (a) Representative images at merged 5X magnification (scale bar = 2 mm) of PSR and enlarged epicardial section at 20X (scale bar = 10um) are displayed. Robust cardiac remodeling and increased fibrosis is more evident in ischemic areas compared to nonischemic and border zone regions. (b) Quantification of collagen content demonstrated significant difference in groups (*p = 0.042) and zones (*p = 0.017), significant difference between remote and infarct zone in MI only swine hearts (**p = 0.0086), and significant difference between border zone in control and MI + HPAC (*p = 0.042). Data is presented as mean ± S.E.M.

Fourteen days after IRI, the pigs were sacrificed, LV punch biopsies were collected, and short‐axis LV slices were stained with TTC to differentiate live/dead tissue (Figure 2a) and H&E to assess cell infiltration. Infarct size was calculated for all slices on both sides and was calculated as a percent fraction of total LV area. HPAC placement on the heart following MI significantly (*p* = 0.039) protected against ischemic injury; HPAC‐treated hearts presented with an infarct size of 11.0 ± 2.0% compared to 22.0 ± 3.0% in MI only hearts (Figure 2b). This was further supported by quantification of collagen content by picrosirius red staining (Figure 3).

**FIGURE 4:**
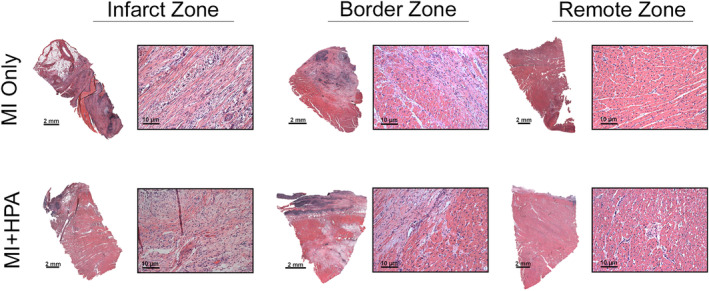
H&E staining revealed changes in swine cellular architecture. (a) LV tissue from infarct, border, and remote zones were stained with Hematoxylin and Eosin (H&E) (*n* = 3 per group), and representative images at merged 5X magnification (scale bar = 2 mm) and 20X (scale bar = 10um) are displayed. Tissue from MI + HPAC infarct zone revealed (b) interstitial hemorrhage (white arrow), (c) granulation tissue with giant cells, (d) disordered cardiomyocytes, and (e) proliferation of lymphocytes and dendritic cells.

H&E staining revealed transitional changes in cellular architecture across IZ, BZ, and RZ regions. Compared with controls, MI induced significant inflammation, including immune cell infiltration, and cardiomyocyte loss in IZ and BZ tissue biopsies. This response was reduced in the IZ of MI + HPAC group compared to MI only group (Figure 4). BZ showed a mosaic pattern with necrotic and granulation tissue in both experimental groups. A higher magnification of the epicardial surface revealed disordered cardiomyocytes, interstitial hemorrhage, and granulation tissue with a proliferation of lymphocytes, giant cells, and dendritic cells in the MI + HPAC IZ group (Figure 4b–e). Vasculature was observed in both groups, often with pericytes skirting the basal lamina. These findings were consistent with the proliferative phase of infarct healing and initiation of angiogenesis.

### 3.3 MI + HPAC infarct zone exhibits ongoing immune response

**FIGURE 5:**
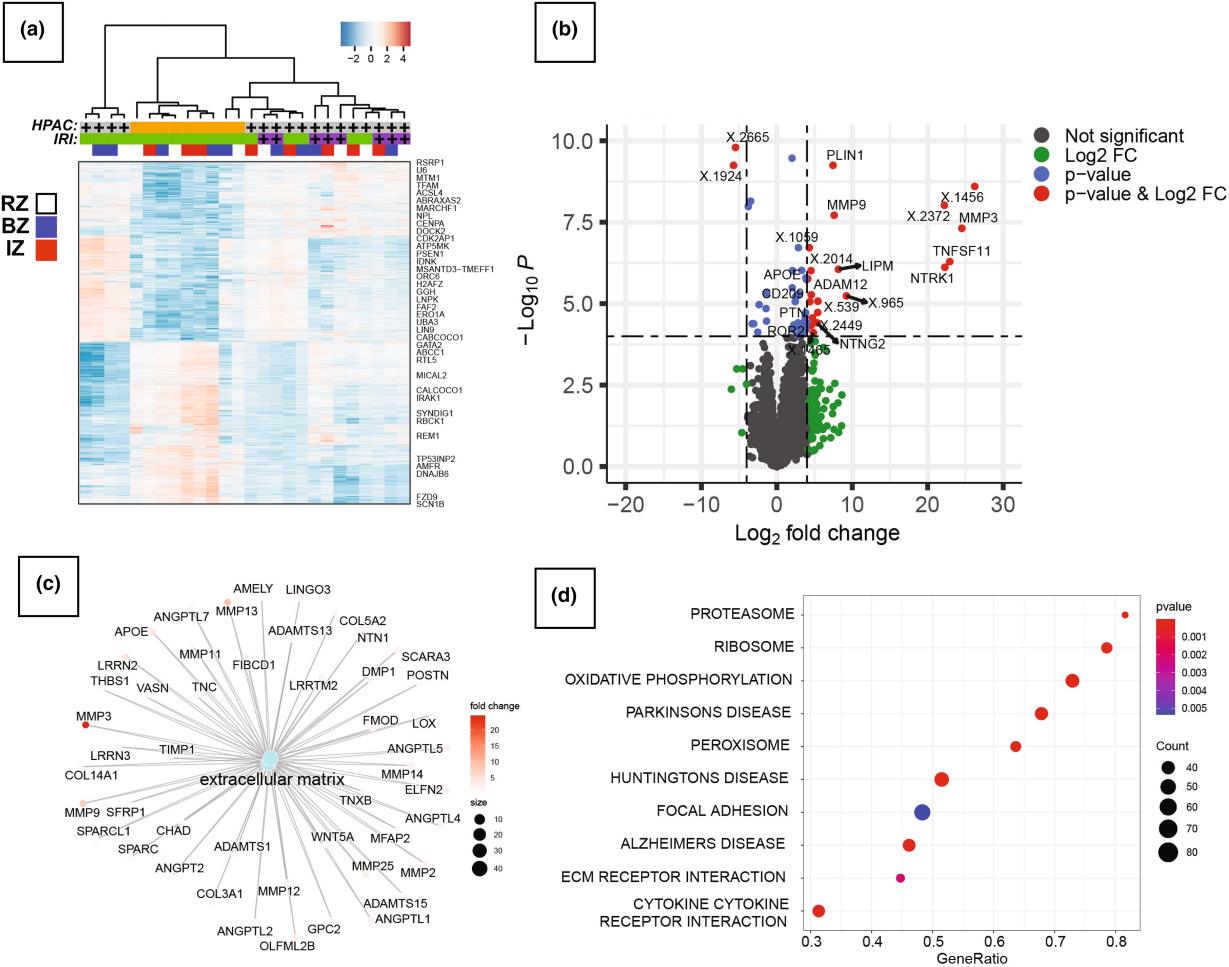
Transcriptome profile of punch biopsies from MI + HPAC and MI only hearts. (a) Heatmap representation of the transcriptome analysis with differentially expressed genes. High to low expression is shown by a degradation color from red to blue, respectively. The scale bar shows Z‐score values for the heatmap. (b) Volcano plot of ‐log_10_ (*p* value) versus log_2_ fold change. The ‐log_10_ (p values) represents the level of significance of each gene while log_2_ fold change represents the difference between the levels of expression for each gene between MI + HPAC and MI only groups. (c) KEGG pathway network analysis of upregulated genes (*p* < 0.05) in MI + HPAC versus MI only hearts. The functionally grouped network was visualized based on the degree of connectivity (node size) to extracellular matrix (ECM) pathways. (d) KEGG pathway enrichment bubble plot. The ratio of the number of different proteins in the corresponding pathways to the total number of proteins identified in the graph is greater, indicating the higher difference in protein concentration in the pathway. The size of the dots represents the number of different proteins in the corresponding pathway and the greater the difference in the pathway represents the greater number of proteins.

To investigate the transcriptional changes underlying phenotypic differences due to HPAC tissue, we performed RNA sequencing on LV punch biopsies from IZ, BZ, and RZ regions. To visualize the transcriptomic relationship between treatment groups and specific regions, a heatmap was generated showing |log_2_ (fold‐change)| > 2 (Figure 5a). Unsupervised hierarchical clustering illustrated IZ from HPAC‐treated (MI + HPAC) and untreated (MI only) hearts was, as expected, closer in distance compared to controls and non‐infarcted (BZ and RZ) regions. A direct comparison of HPAC‐treated versus untreated hearts using a volcano plot of ‐log_10_ (p values) versus log_2_ fold change indicates significant increases dominated by transcripts targeting matrix metalloproteinase (MMP) genes (*MMP9* and *MMP3*) (Figure 5b). Subsequent pathway network analysis revealed an overwhelming enrichment of differentially regulated genes for extracellular matrix, components of which are the major targets of the MMP family of proteins (Figure 5c). In the KEGG database, 10 pathways were substantially enriched (*p* ≤ 0.05), including focal adhesion, cytokine‐cytokine receptor interaction, and oxidative phosphorylation in the MI + HPAC response compared to MI only (Figure 5d). Specifically, C‐C and C‐X‐C motif chemokine ligands, *IL‐1β*, *IL‐17*, *CSF2RB*, and *OSM* were upregulated. In addition, JAK–STAT signaling was found to be downregulated through *EPO*, *IFN*, and *IL2*/3 and cytokine receptors, resulting in the downregulation of cell cycle and anti‐apoptosis. Chemokine signaling pathway noted upregulation of chemokines led to the downregulation of *PI3K* and downstream ubiquitin‐mediated proteolysis.

### 3.4 HPAC modulates inflammatory and extracellular matrix pathways

**FIGURE 6:**
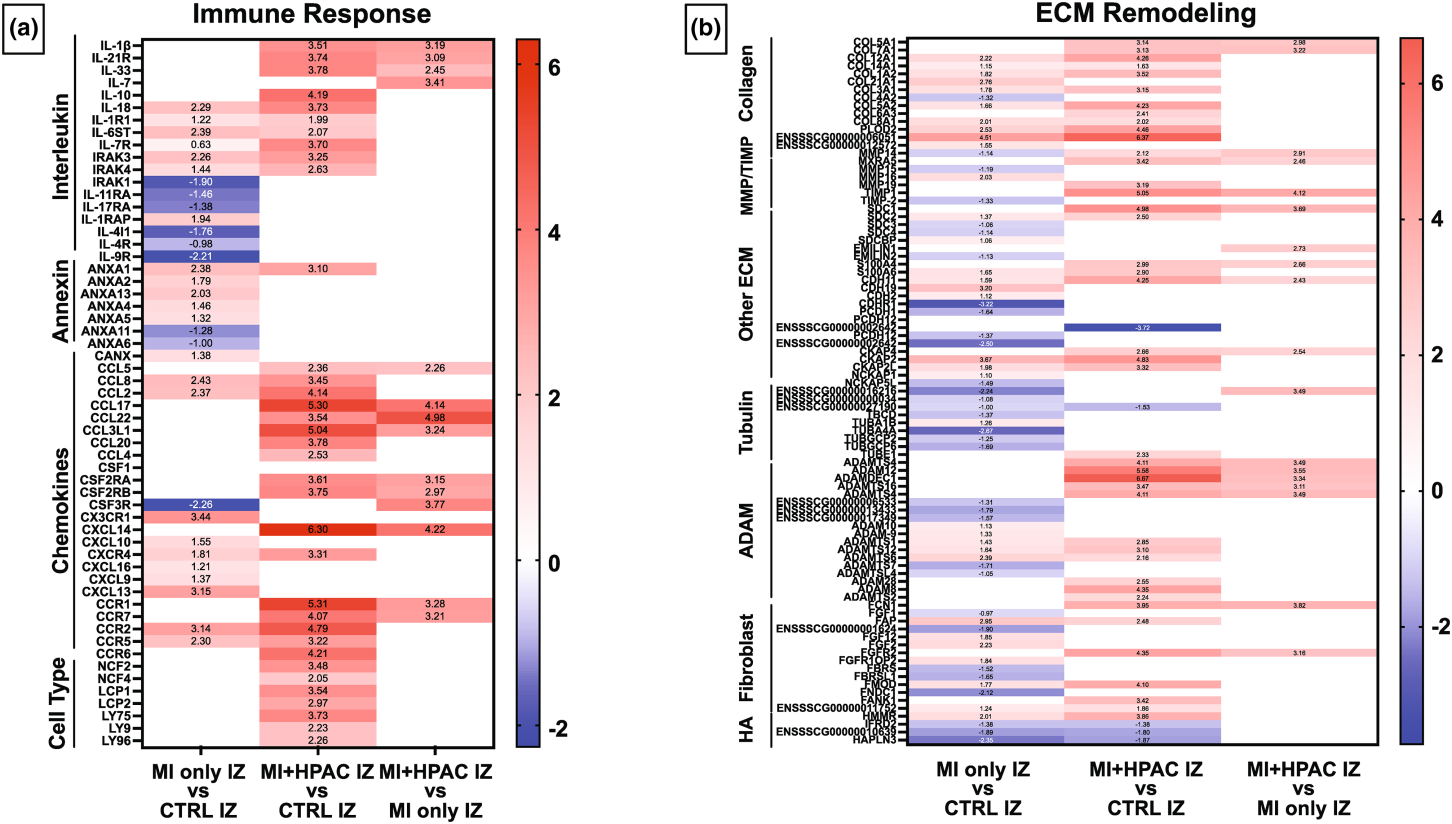
Immune and ECM profiles alter with HPA treatment. (a) Heatmap of differential expressed immunomodulatory genes between MI only IZ versus control IZ, MI + HPAC IZ versus control IZ, and MI + HPAC IZ versus MI only IZ. (b) Heatmap of differential expressed ECM genes between MI only IZ versus control IZ, MI + HPAC IZ versus control IZ, and MI + HPA IZ versus MI only IZ. HPAC use demonstrates wound healing response. *N* = 3 for each group.

Further interrogation of IZ profiles of MI + HPAC and MI only depicted a 2–3‐fold increase in the interleukin genes *IL‐1β* (p‐adj 0.023), *IL‐21R* (p‐adj 0.017), *IL‐33* (p‐adj 0.045), and *IL‐7* (p‐adj 0.0082) with MI + HPAC treatment (Figure 6a). Additionally, inflammatory chemokine receptors associated with neutrophils and adaptive immune cells, including *CCR1* (3.3, p‐adj 0.0085), *CCR7* (3.2, p‐adj 0.016), respectively, were upregulated. *CCR2*, a leukocyte chemoattractant and known mediator in CAD, demonstrated a 3‐fold increase (p‐adj 3.6E‐04) in MI only IZ and 4.8‐fold increase (p‐adj 8.2E‐05) in MI + HPAC compared to control IZ. However, this variation was not statistically significant between experimental groups, suggesting the gene is induced by the MI response. Although no direct neutrophil markers were found to be significant in MI + HPAC IZ, *LCP1*, an actin‐binding protein that regulates T cell activation, was upregulated when compared to control IZ (3.54, p‐adj 6.8E‐05) and MI only IZ (2.08, p‐adj 0.081).

We also identified trends in specific anti‐inflammatory biomarkers upregulated by HPAC treatment in the IZ. Although there was a 4.2‐fold increase in *IL‐10* (p‐adj 0.0015) in MI + HPAC IZ compared to control IZ, induction of *IL‐10* in the MI only IZ, compared to the HPAC treatment, was not significant (1.79, p‐adj 0.31). The same trend with other anti‐inflammatory markers including *IL‐6ST*, *IL‐4I1*, and *TGFβ*, was evident. *ANXA‐1*, an anti‐inflammatory and apoptotic mediator, was upregulated in both MI only (2.4, p‐adj 2.2E‐06) and MI + HPAC (3.1, p‐adj 3.1E‐04) compared to control IZ; however, direct comparison of experimental groups did not show significant difference (0.69, p‐adj 0.85).

To evaluate the impact of HPAC treatment on cardiac remodeling, ECM profiles of IZ tissues between control, MI only, and MI + HPAC were compared (Figure 6b). A 3‐fold increase in *COL5A1* (p‐adj 0.011) and *COL7A1* (p‐adj 0.0078) was seen in MI + HPAC group IZ compared to MI only IZ. Similarly, *MMP14* (2.91, p‐adj 0.0055), *ADAM*, and *ADAMTS* involved in degrading and rebuilding matrix are upregulated in MI + HPAC IZ with respect to MI only IZ. This response may be modulated by the upregulation of *TIMP1* (4.1, p‐adj 5.7E‐04), an ECM proteolysis inhibitor. Other matrix proteins including *SDC1* (3.7, p‐adj 0.0013), *S100A4* (2.7, p‐adj 0.015), *EMILIN1* (2.7, p‐adj 0.014), and *CDH11* (2.4, p‐adj 0.049) were altered with HPAC treatment. Taken together, we identified an active inflammatory response coupled with significant ECM pathway activity in the IZ of MI + HPAC hearts.

### 3.5 HPAC induces molecular changes in the remote zone

The importance of deciphering the molecular modifications within the RZ and its influence in remodeling cannot be understated. Therefore, we compared the RZ within experimental groups. The wound healing responses in the MI + HPAC RZ sample compared to control RZ (Figure S3) were distinct from the MI only RZ responses compared to control RZ. This indicates that the HPAC has global cardiac effects outside the actively remodeling IZ and BZ regions. Moreover, pathways involved in collagen trimer assembly and striated muscle cell differentiation were apparent in the MI + HPAC RZ tissue. When RZ was compared to IZ of untreated and HPAC‐treated hearts, specific changes in proteins associated with protein trafficking, autophagy, and angiogenesis were impacted (Figure S4). Consequently, changes noted in the RZ suggest future work should further define alterations in signature profiles with HPAC placement in other zones.

### 3.6 HPAC mitigates LV hypertrophy and promotes decrease protein secretion

**FIGURE 7:**
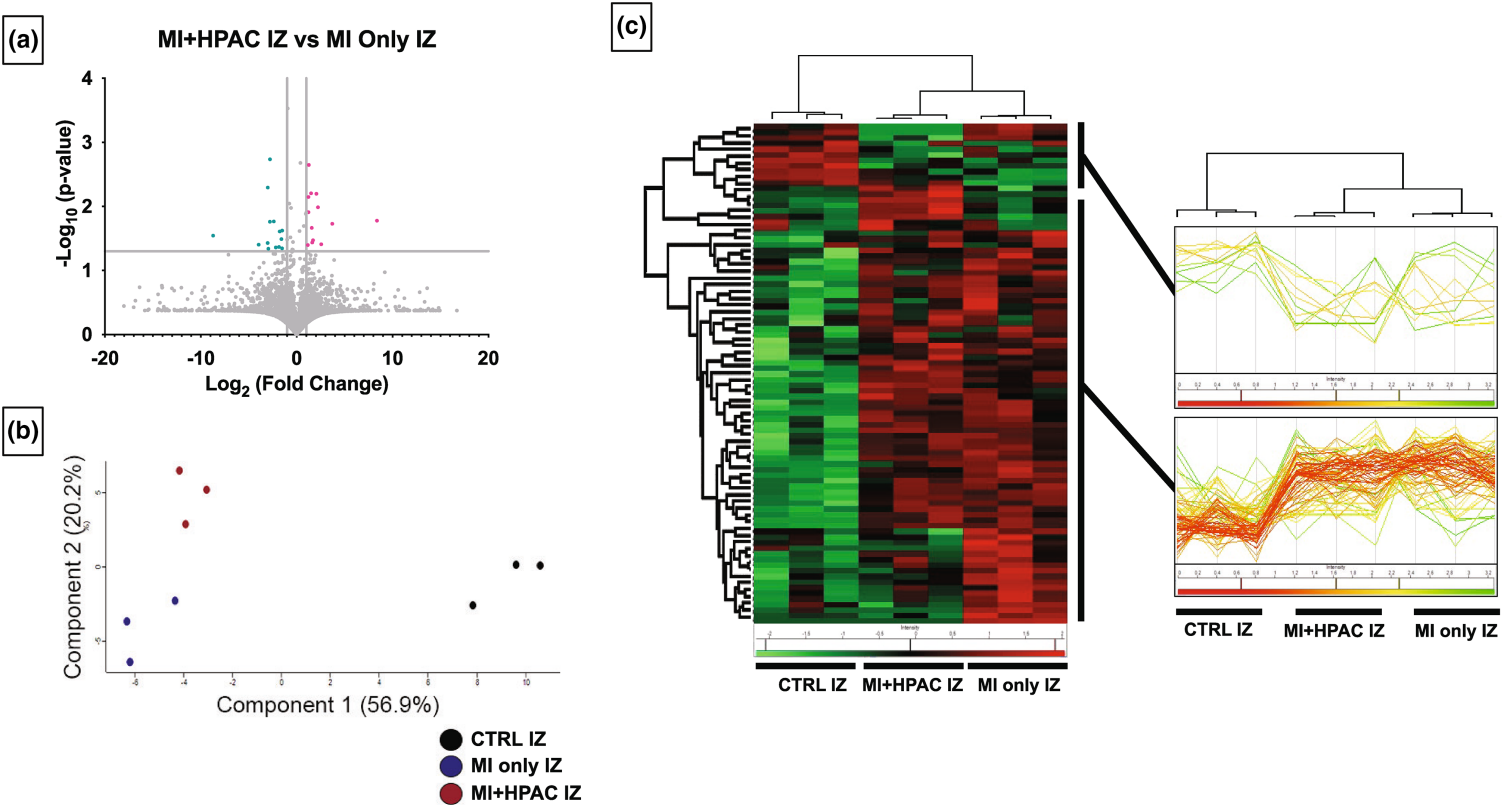
Proteomic analysis of infarct zone. (a) Volcano plot of MI + HPA infarct zone (IZ) to MI only IZ show 13 upregulated [pink] and 14 downregulated [teal] proteins as ‐log_10_(p‐value) and log_2_(fold change). (b) Principal component analysis of IZ of all three groups with PC1 of 56.9% and PC2 of 20.2%. (c) Statistical clustering and visualization using Hierarchical clustering (heat map) of IZ from MI + HPAC and MI only hearts. Expression levels were color coded, and clusters highlighted in the dendrogram and Z‐score weighted clusters showed distinct expression patterns visualized as a heat trace. *N* = 3 per group. *p* < 0.05 using one‐way ANOVA.

Proteomic analysis of porcine “punch biopsies” from MI only and MI + HPAC groups yielded approximately 5300 hits demonstrating robust proteome coverage (Figure 7). The volcano plot directly comparing IZ from MI + HPAC and MI only hearts identified 13 upregulated and 14 downregulated proteins (Figure 7a). Principal component analysis of IZ from MI + HPAC and MI only groups including controls all three groups with PC1 of 56.9% and PC2 of 20.2%. Using Perseus software platform (http://www.perseus‐framework.org) (Tyanova et al., 2016), proteomic data from IZ of MI + HPAC and MI only hearts was statistically clustered and visualized using hierarchical clustering (heat map). Expression levels were color coded and clusters highlighted in the dendrogram (Figure 7c, left panel). Z‐score weighted clusters showing distinct expression patterns were visualized as a heat trace (Figure 7c, right panel). The heat map clearly indicates two key findings: (1) individual samples denoted by columns cluster (computed distances) more closely (most similar) within each experimental group and (2) MI + HPAC IZ tissue clustered more closely to control IZ than MI only IZ clustered to control IZ.

**FIGURE 8:**
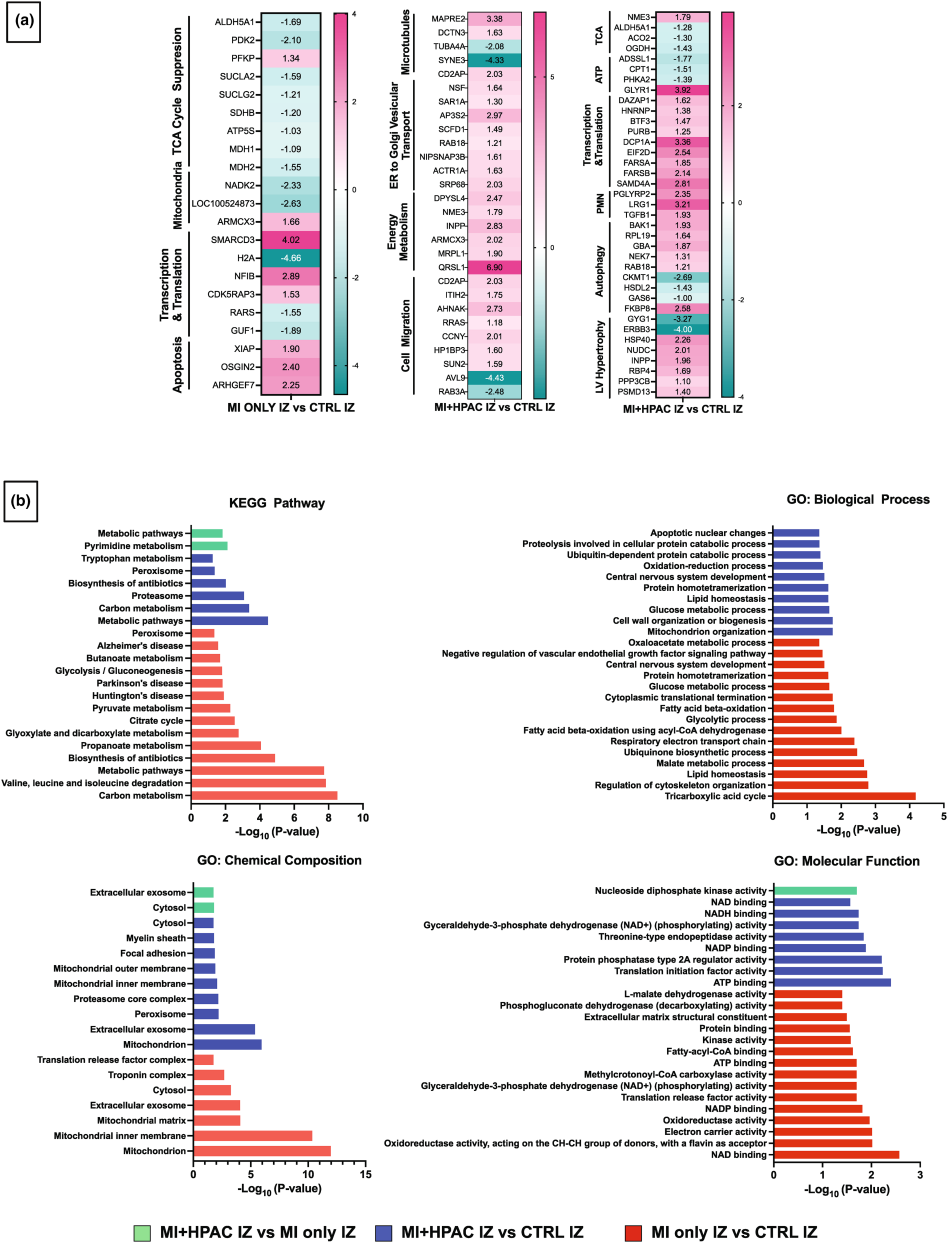
Proteomic analysis of infarct zone versus non‐infarct tissue. (a) Mini‐heatmaps of differentially expressed proteins between MI only IZ versus control IZ (non‐infarct) and MI + HPAC IZ versus control IZ. (b) GO and KEGG analysis of top pathways of the differentially expressed proteins in IZ between all three groups.

For MI + HPAC IZ versus MI only IZ, changes in mitochondria (*CCDC51*, 1.57, *p* = 0.022), lipid metabolism (*HACL1*, −2.4, *p* = 0.017), transcription (*NOVA2*, 2.6, *p* = 0.039) and translation (*PUS1*, 2.1, *p* = 0.0063) regulation resulted in an overall decrease in protein secretion (*DNAJA2*, *BTF3*, *STXBP6*) and increase in apoptosis (*HTATIP2*, 1.5, *p* = 0.045). Hypertensive and volume‐overload responses following IRI appear to be mitigated by the HPAC, with inhibition of renin activity (*RENBP*, 1.5, *p* = 0.0063) and mechanosensitive signaling (*LRRC10*, 3.0, *p* = 0.0051). Moreover, *MARK3* (−3.0, *p* = 0.045), a Class IIa HDAC kinase, was downregulated, indicating active prevention of hypertrophy. Consistent with RNAseq findings, proteomic analysis corroborated mechanisms involving *MAPKAPK2*, *PLCD1*, *AKT2*, and *TNIP1*. Interestingly, mini‐heatmaps of differentially expressed proteins between MI only IZ versus control IZ and MI + HPA IZ versus control IZ indicated limited overlap in the IZ among MI and MI + HPAC treated hearts (Figure 8). GO and KEGG pathways analysis indicated a similar pattern of differentially expressed proteins in IZ between all three groups.

### 3.7 HPAC may protect the heart from IRI through activation of heat shock proteins

**FIGURE 9:**
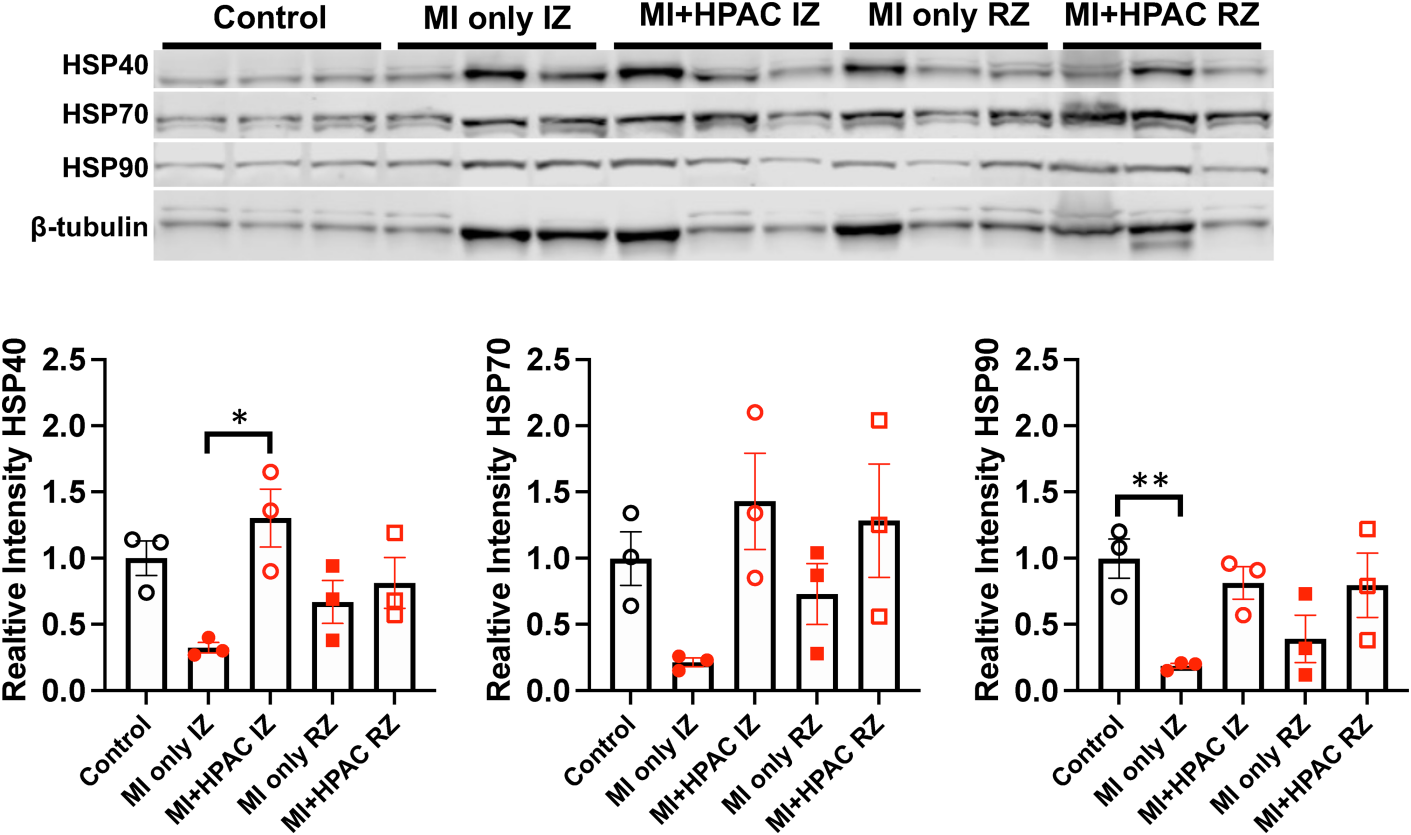
Immunoblot Analysis of Heat Shock Proteins in Control, IZ, and RZ with and without HPAC. Top Panel: Western blot depicting protein expression of total HSP40, HSP70, HSP90, and β‐tubulin. Bottom Panel: Bar graph summary of HSP40 (left), HSP70 (middle), and HSP90 (right) comparing protein levels in CONTROL, MI only IZ, MI + HPAC IZ, MI only RZ, and MI + HPAC RZ. Total HSP40, HSP70, and HSP90 were normalized to β‐tubulin after adjusting for total protein determined from Ponceau S. Data presented as mean ± S.E.M; *n* = 3 for each group. HSP40 and HSP90 levels were significantly different (*p* = 0.0159 and *p* = 0.0264, respectively, by ordinary one‐way ANOVA and the Tukey post hoc test; *p = 0.0103, **p = 0.0320 by post‐hoc analysis).

The extreme metabolic and oxidative stress induced by IRI triggers the release of heat shock proteins (HSP/*DNAJA2*), which assist in the removal of damaged proteins, inhibit apoptosis, and maintain the structural integrity of the sarcomere. (Ranek et al., 2018) Accordingly, porcine LV “punch biopsies” were immunoblotted from members of the HSP signaling axis including HSP40, HSP70, and HSP90 (Figure 9). Although no significant differences were detected in HSP70 levels (*p* = 0.0820), HSP40 and HSP90 levels were significantly impacted (*p* = 0.0159 and *p* = 0.0264, respectively) by IRI and HPAC treatment. More specifically, HSP40 levels in the MI only IZ were significantly (*p* = 0.0103) attenuated by HPAC treatment (MI + HPAC IZ, *p* = 0.0103) while HSP90 levels were significantly (*p* = 0.0320) reduced by IRI compared to control hearts (MI only IZ vs. Control). Importantly, there were no significant differences between MI + HPAC IZ, Control, MI only IZ, or MI + HPAC RZ suggesting placement of HPAC protected against ischemic injury.

## 4 DISCUSSION

Cardiac repair following MI is initiated by a robust and coordinated inflammatory charge characterized by chemical signals and recruitment of distinct populations of inflammatory cells. (Nahrendorf et al., 2007; Panizzi et al., 2010) Previous studies suggest the utility of acellular bioscaffolds in promoting cardiac repair following MI potentially by promoting proangiogenic and antifibrotic pathways. (Svystonyuk et al., 2020; Vasanthan et al., 2020; Vasanthan et al., 2021) The current study demonstrated that application of HPAC membranes to swine hearts post‐MI attenuates MI expansion and promotes MI repair, key beneficial indicators to prevent HF. Adjunctive treatment with HPAC in other medical conditions, such as diabetic ulcers and ligament repairs, implicates a beneficial utility for wound healing. We were the first group to recognize the clinical application of HPAC tissue grafts following revascularization of an ischemic myocardium in humans. (Avery et al., 2018) HPAC has had promising results in reducing postoperative atrial fibrillation and postoperative inflammation, a common complication in cardiac surgery. Given positive results in animal model in this study, employing HPAC grafts during coronary artery bypass graft procedures would temper the inflammatory effects, thereby promoting long‐term benefit and better clinical outcomes. (Khalpey et al., 2015; Marsh et al., 2017; Rao et al., 2018) Although it is likely that the underlying mechanism can be attributed to HPAC being immunologically privileged and retaining the endogenous properties of the natural tissue, the specific cellular and molecular pathways remain undefined. (Khalpey et al., 2015; Marsh et al., 2017).

We evaluated the clinically relevant swine IRI model to elucidate the cellular and molecular mechanisms of HPAC membrane‐induced cardioprotection. Considering the complex interplay between bioscaffolds and underlying ischemic cardiac tissue, a deep phenotyping strategy was used to spatially interrogate the cellular and molecular mechanisms during the proliferative cardiac repair phase (14 days) following MI. (Svystonyuk et al., 2020; Vasanthan et al., 2020; Vasanthan et al., 2021).

We present the first comprehensive transcriptome‐proteome comparison matched with physiological and histological phenotyping in a non‐rodent, large animal model of IRI with and without HPAC membrane treatment. HPAC placement following MI provided measurable benefit indicated by key clinically relevant parameters including reduction of cardiac damage, preservation of EF, and maintenance of ventricular chamber and wall dimensions when compared to MI controls. MI only swine exhibited decreased EF, dilated cardiac remodeling, and volume overload consistent with post‐MI remodeling and rapid progression to HF. (Agnew et al., 2019) Functional and morphological differences between HPAC treated and untreated hearts was supported by histological assessments. (Rusinkevich et al., 2019; Yan et al., 2013).

Transcriptomic analysis revealed a significant immune response. Specifically, cytokine and chemokine markers indicated lymphocyte, macrophage, and dendritic infiltration of the heart with HPAC treatment. This pattern of immune cells is consistent with the proliferative phase of cardiac repair as noted in other studies. (Matsumoto et al., 2011) Again, histological evidence by H&E supported transcriptome findings as demonstrated by macrophage and adaptive immune cell influx. Transcriptome analysis also revealed a prolific cytokine and chemokine response, including *IL‐1β*, *IL‐21R*, *IL‐33*, *and IL‐7* with HPAC treatment. Potent immune regulators such as IL‐1β are mediated by inflammasomes and often induced by pro‐inflammatory monocytes following ischemia. They serve to regulate MMP expression, apoptosis, phagocytosis, and adhesion molecule activation. A chemokine, specifically *CCL5*, was upregulated with HPAC treatment, and is known to help determine infarct size and HF. (Khanian et al., 2016; Montecucco et al., 2012) These data indicate a robust and active immune response in the IZ of HPAC‐treated hearts compared to untreated, MI only hearts.

Proteomic analysis was highlighted by an over‐abundance of mediators dictating the extent and type of cardiac remodeling post‐MI. The PI3K/Akt/mTOR signaling pathway regulates protein synthesis, and activation of PI3K ultimately phosphorylates mTOR to inhibit apoptosis proteins such as caspases and promote antiapoptotic factors including Bcl‐2. Other studies concur with our transcriptome data that this pathway is downregulated with IRI. (Liao et al., 2019) Proteomic analysis suggests that HPAC treatment promotes controlled cell death with apoptosis induced by ER‐stress and autophagy. It is not clear how apoptosis enhances cardiac regeneration, but we speculate that removal of compromised cells may be important.

Increased collagen deposition coupled with MMP turnover denote fibrosis and matrix rearrangement following IRI. This reorganization protects from cardiac rupture and favors cardiac function, as evidenced by echocardiography. Our data suggest the efficacy of HPAC membrane may act as a conduit for immune cell infiltration and removal of dead cells and debris while providing a more ideal scaffold for ECM remodeling. The strikingly selective localization of ECM proteins suggests a complexity of protein function beyond matrix remodeling. Temporal regulation of ECM with stop signals for proliferation is vital to prevent ventricular stiffness and diastolic dysfunction. Hence, the balance of proliferation and degradation with targeted spatial regulation is essential.

Another striking pattern identified was altered protein secretion with HPAC application. Proteome profiles of IZ samples demonstrated decreased ER‐dependent protein secretion and targeting. This coupled with less targeted ubiquitination can result in reduced clearance and protein aggregation. Nevertheless, other studies suggest that proteasome inhibition seems to confer cardioprotection through NF‐ĸB inhibition and regulation of heat shock proteins. (Zolk et al., 2006) Hsp40 serves as a modulator and co‐chaperone for Hsp70 to stimulate ATP hydrolysis. Previous studies have demonstrated Hsp40 is sensitive to ischemic processes and have revealed pDHA1, the Hsp40 transcript, reduces irreversible damage in in vitro models. As such, elevated levels can promote metabolic improvements and contribute to cardioprotective effects. (Willis & Patterson, 2010) Identification of which protein levels are maintained, accumulated, and degraded will determine the beneficial response observed with HPAC.

In the RZ myocardium, cardioprotective anti‐apoptosis, negative regulation of proteolysis, and macrophage polarization dominated the proteomic signature in HPAC‐treated hearts. Collagen deposition and hypertrophic proteins were upregulated perhaps indicating a dynamic transition of compensatory hypertrophic growth to preserve cardiac function.

In summary, we find that use of HPAC membrane in this swine animal model, immediately after infarct, provides an improved outcome in preservation of function and remodeling of the tissue. We also provide evidence that transcriptomic and proteomic data both suggest continued activity of immune cell mediators, protein secretion, and ECM remodeling which underlie a molecular and cellular basis for HPAC‐dependent cardioprotection. The translational relevance and potential for therapeutic development relies more heavily on positive functional and morphological outcomes. However, understanding of HPAC efficacy and identification of more precise clinical indications will ultimately be revealed from integration of data matrices. Only through combinatorial, deep phenotyping approaches will we provide fundamental new knowledge of mechanisms mitigating IRI, while providing translational insight to elucidate the full potential of HPAC in MI.

## FUNDING INFORMATION

Gift to the University of Arizona Sarver Heart Center from MTF Biologics.

## CONFLICT OF INTEREST STATEMENT

Dr. Zain Khalpey—Paid consultant for MTF Biologics.

## ETHICS STATEMENT

All experiments were performed using protocols that adhered to guidelines and approved by the Institutional Animal Care and Use Committee at the University of Arizona and to 2019 NIH guidelines for care and use of laboratory animals.

## Supporting information


Figure S1:

Figure S2:

Figure S3:

Figure S4:

Figure S5.

Figure S6.

**Data S1:** Supporting Information
