# Supplementary figures and images for: Epicardial placement of human placental membrane protects from heart injury in a swine model of myocardial infarction
Source: Physiol Rep. 2023 Oct 17;11(20):e15838. doi: 10.14814/phy2.15838 (PMC10582231; doi:10.14814/phy2.15838)

**Figure S1.**

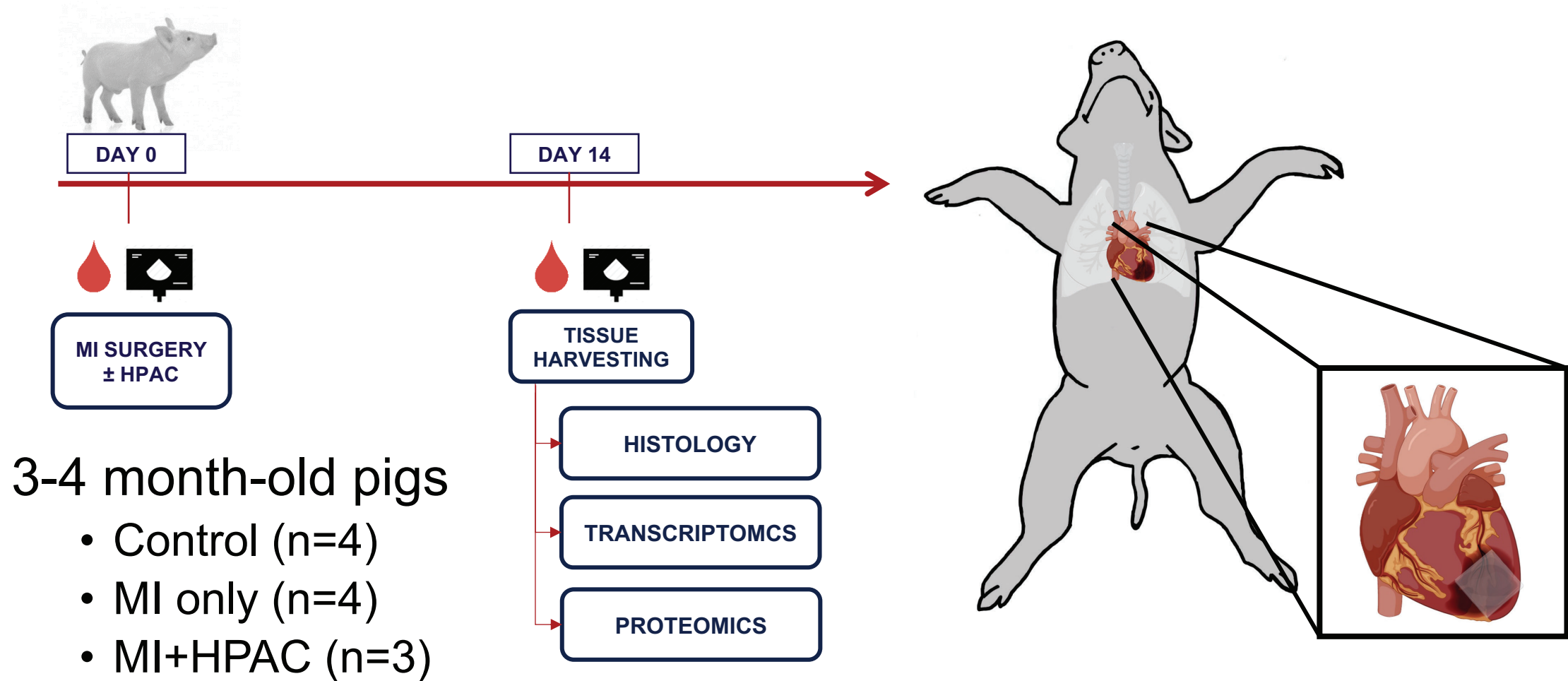

Supplement: Supplementary file 1 — Figure S1: [file PHY2-11-e15838-s002.pdf]

**Figure S2.**

Methods

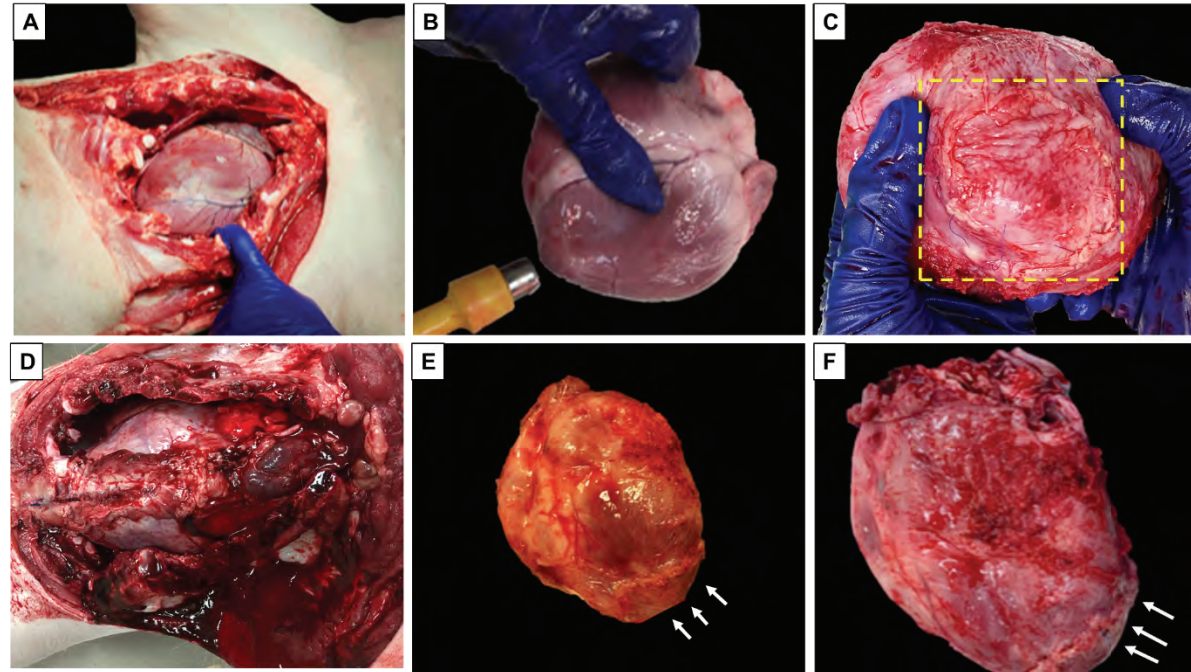

Supplement: Supplementary file 2 — Figure S2: [file PHY2-11-e15838-s003.pdf]

**Figure S4.**

**A**

**MI only IZ vs MI only RZ**

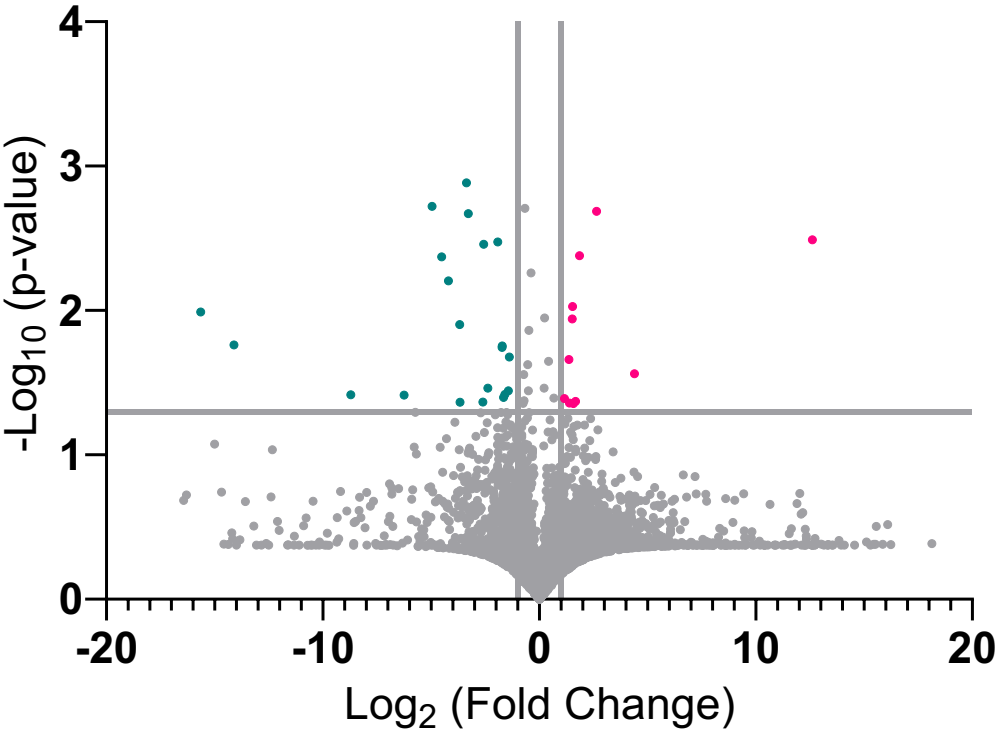

**B**

**MI+HPAC IZ vs MI+HPAC RZ**

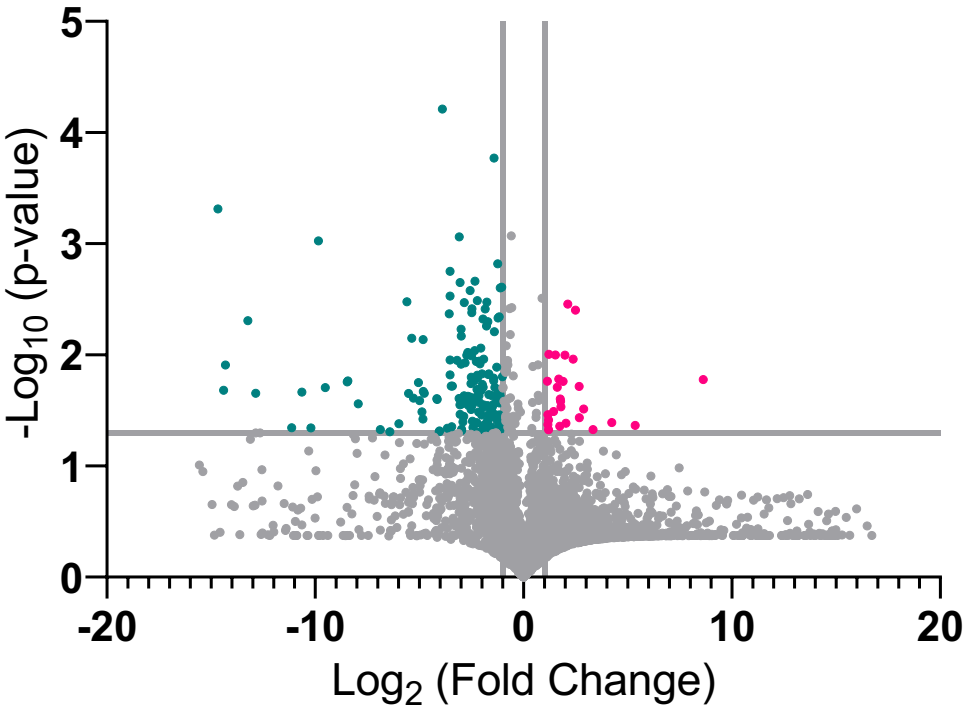

Supplement: Supplementary file 4 — Figure S4: [file PHY2-11-e15838-s001.pdf]

**Figure S5.**

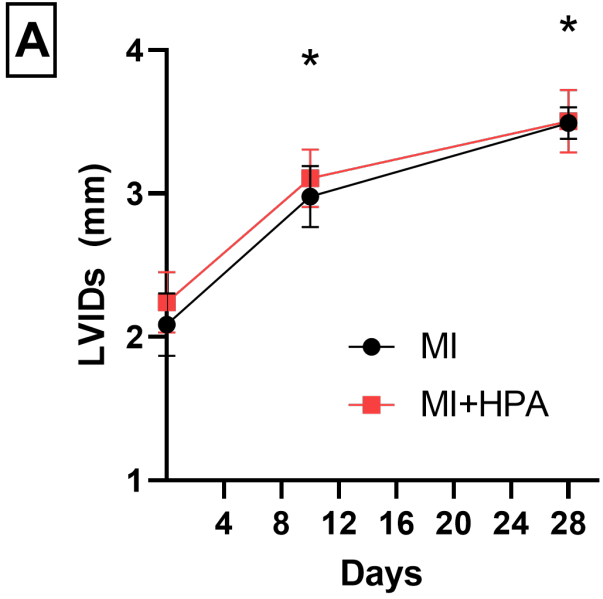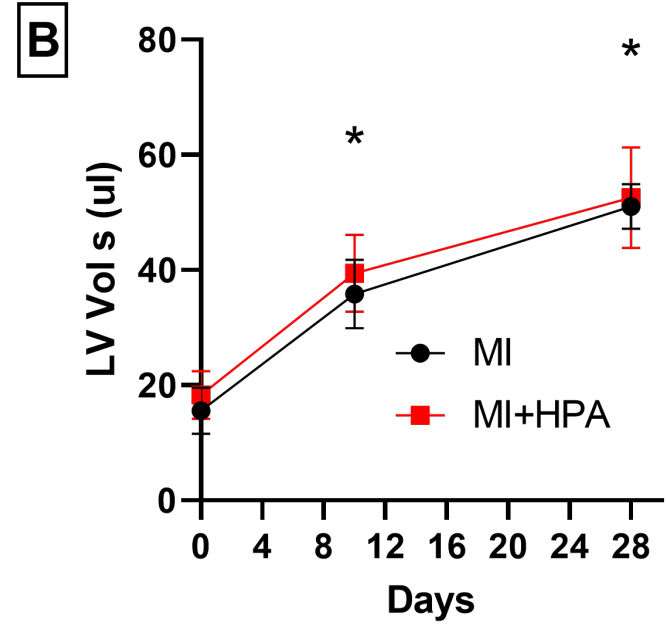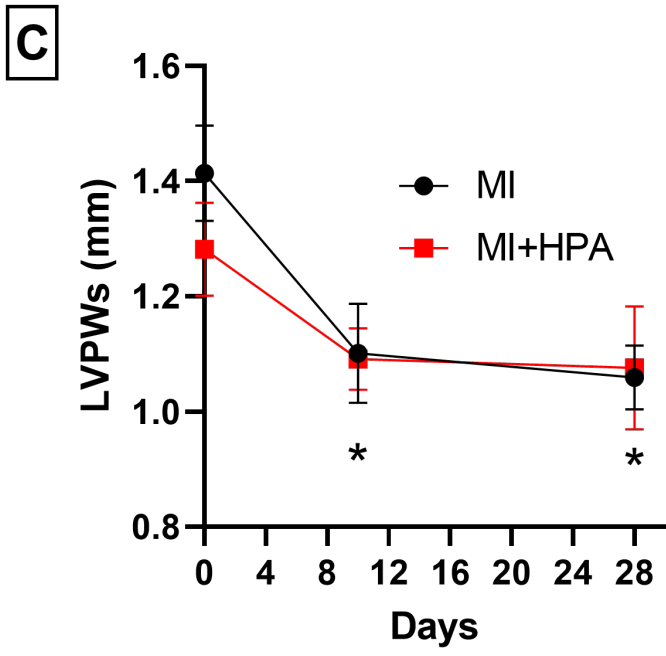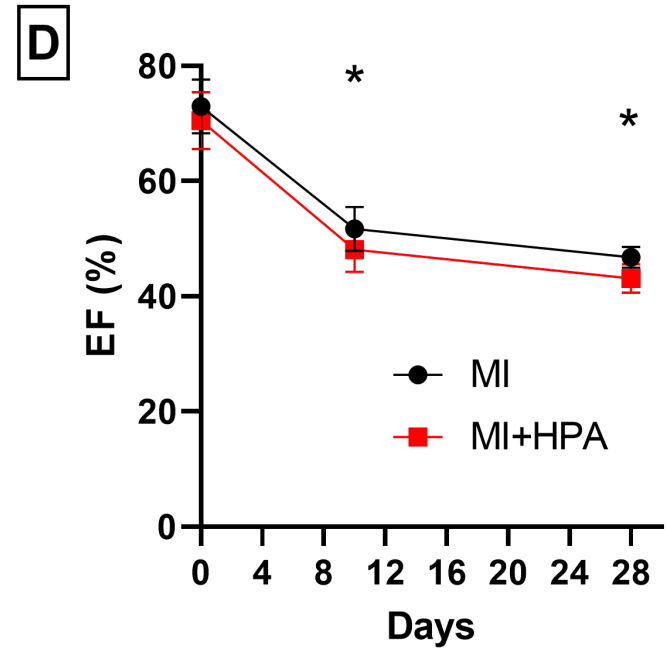

Supplement: Supplementary file 5 — Figure S5. [file PHY2-11-e15838-s006.pdf]

**Figure S6.**

**A**

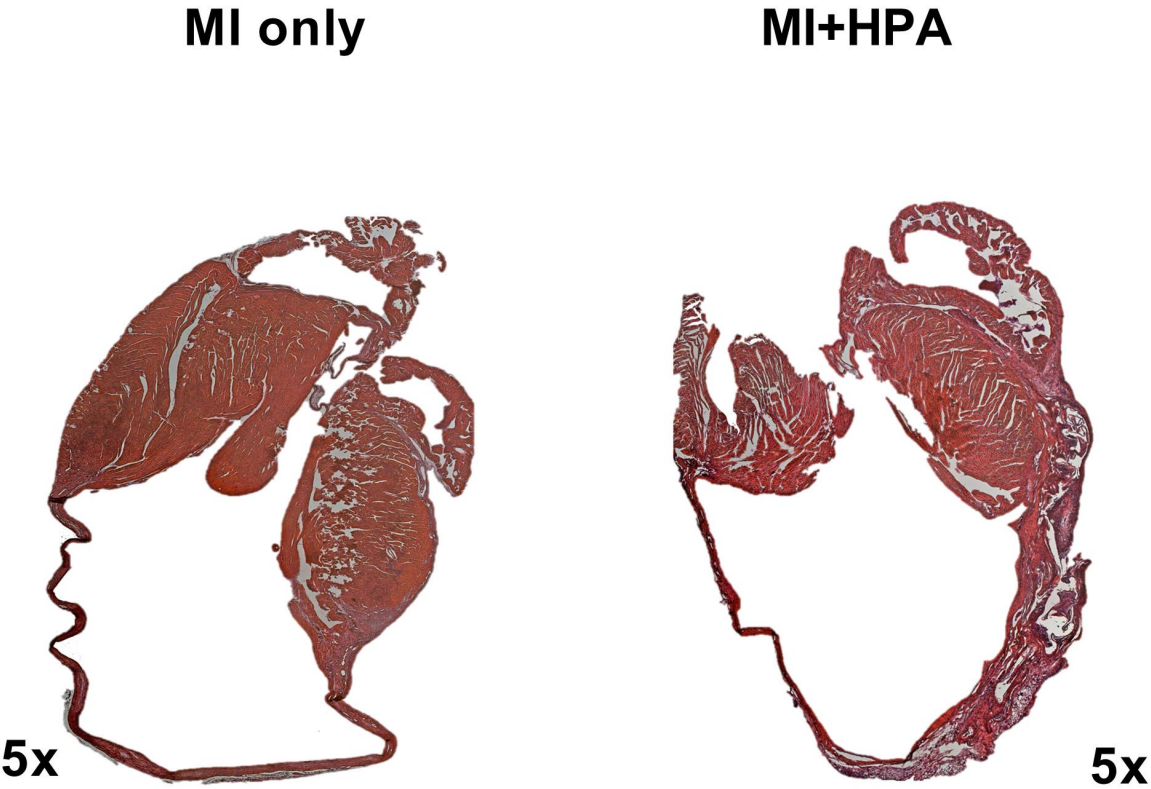

**B**

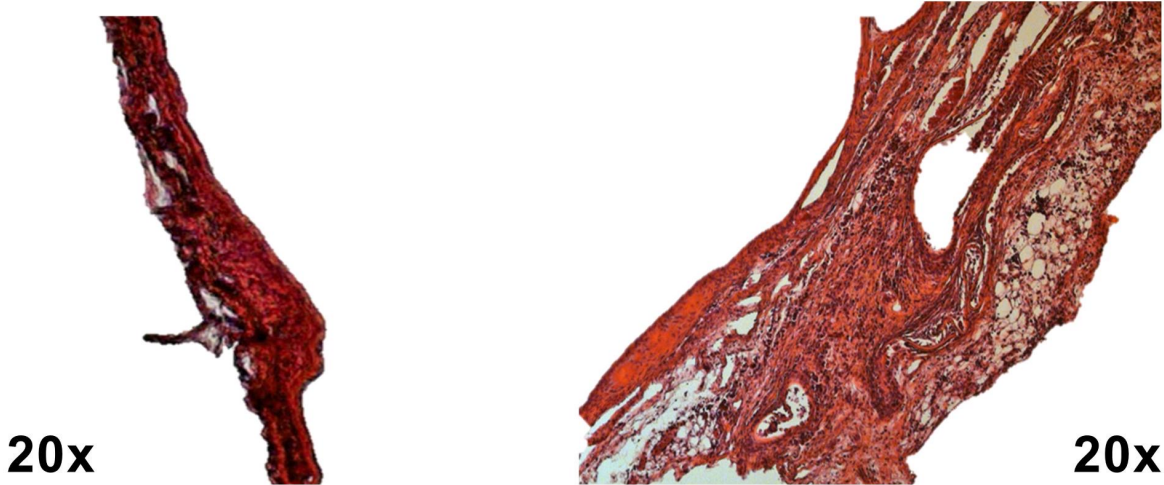

**C**

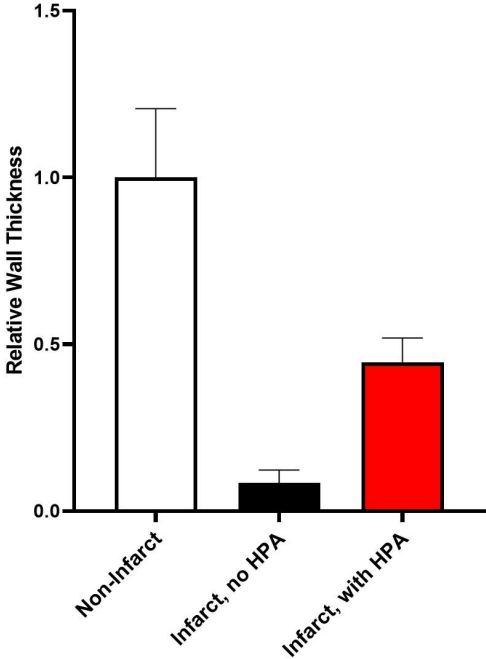

Supplement: Supplementary file 6 — Figure S6. [file PHY2-11-e15838-s005.pdf]
