# Supplementary material for: Epicardial placement of human placental membrane protects from heart injury in a swine model of myocardial infarction
Source: Physiol Rep. 2023 Oct 17;11(20):e15838. doi: 10.14814/phy2.15838 (PMC10582231; doi:10.14814/phy2.15838)
Supplement: Supplementary file 3 — Figure S3: [file PHY2-11-e15838-s007.pdf]

Figure S3

MI+HPAC RZ vs CTRL RZ

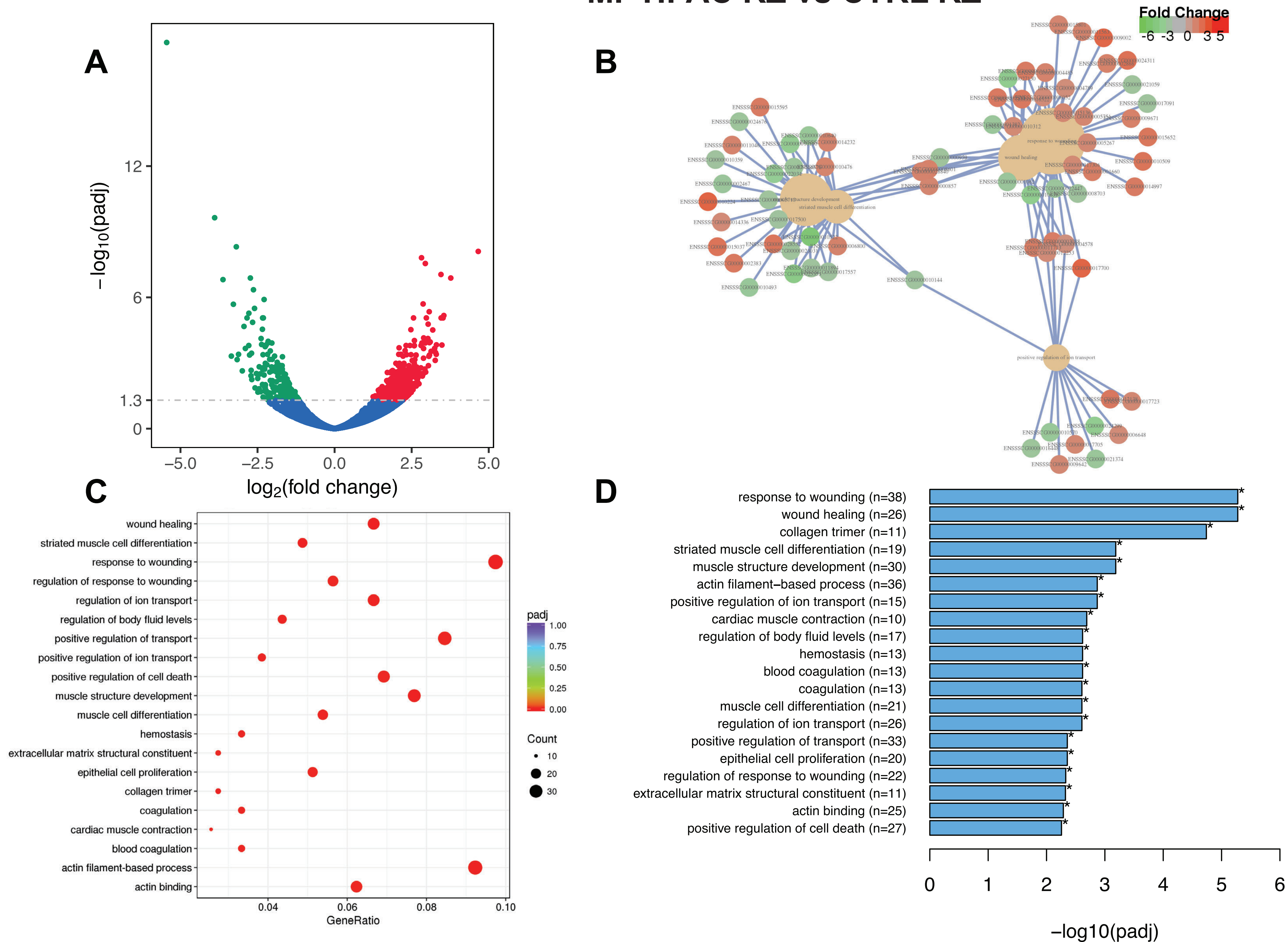

E

Table: Wound Healing

Gene	Log_2(Fold Change)	P-adj Value
TNFRSF12A	-2.2872	1.2518E-06
TLR4	2.2491	0.0023759
PTK7	2.1293	0.0030557
DDR	-1.5967	0.0032851
CD109	1.9	0.0085176
APOE	2.1263	0.013196
EZH2	1.9001	0.013542
DTNBP1	-1.5837	0.013678
PLAU	1.6841	0.016425
ANXA2	1.4193	0.017483
IGF1	1.8084	0.017914
ANXA1	1.7105	0.022221
P2Y12R	2.145	0.023555
CD61	1.697	0.02412
SDC1	1.9853	0.029115
LARGE	-1.4195	0.029115
COL5A1	1.807	0.029799
UBASH3B	1.7585	0.034996
THBS1	1.5177	0.035532
PTN	2.3093	0.037443
F3	-1.5191	0.042392
CASK	1.6137	0.043143
ADRA2C	-1.3578	0.043275
FN1	2.2315	0.044061
FZD6	1.5958	0.049033
