# Supplementary material for: Epicardial placement of human placental membrane protects from heart injury in a swine model of myocardial infarction
Source: Physiol Rep. 2023 Oct 17;11(20):e15838. doi: 10.14814/phy2.15838 (PMC10582231; doi:10.14814/phy2.15838)
Supplement: Supplementary file 7 — Data S1: Supporting Information [file PHY2-11-e15838-s004.docx]

# SUPPLEMENTAL MATERIAL

**Title:** Epicardial Placement of Human Placental Membrane Protects from Heart Injury in a Swine Model of Myocardial Infarction

**Short Title:** Epicardial Placement of Human Placental Membrane

**Authors:** Rinku S. Skaria, Ph.D.^1*^, Marissa A. Lopez-Pier, Ph.D.^2^, Brij S. Kathuria^1^, Christian J. Leber^1^, Paul R. Langlais, Ph.D. ^3^, Shravan G. Aras, Ph.D.^4^, Zain I. Khalpey, M.D., Ph.D.^5^, Pamela G. Hitscherich, Ph.D.^6^, Evangelia Chnari, Ph.D.^6^, Marc Long, Ph.D.^6^, Jared M. Churko, Ph.D.^7,8^, Raymond B. Runyan, Ph.D. ^7,8^, John P. Konhilas, Ph.D.^1,2,8*^

^1^Department of Physiology, University of Arizona College of Medicine, Tucson, Arizona.

^2^Department of Biomedical Engineering, University of Arizona College of Engineering, Tucson, Arizona

^3^Department of Medicine, University of Arizona College of Medicine, Tucson, Arizona.

^4^Center for Biomedical and Informatics, University of Arizona Health Sciences, Tucson, Arizona.

^5^Northwest Healthcare, Tucson, Arizona

^6^MTF Biologics, Edison, NJ, United States,

^7^Department of Cellular and Molecular Medicine, University of Arizona College of Medicine, Tucson, Arizona

^8^Sarver Molecular Cardiovascular Research Program, University of Arizona College of Medicine, Tucson, Arizona

**Acknowledgements:** University Animal Care, Dr. Josh Strom, Alice McArthur, Arielle Tran, Christina Hoyer-Kimura, Martha Nunez, Maricela Pier

## Supplemental Methods

### Human Placental Amnion/Chorion Grafts: HPAC graft (AmnioBand® Membrane, MTF Biologics, Edison, NJ) is a dehydrated membrane comprised of both amnion and chorion layers. All tissues from MTF were aseptically recovered and processed and receive an in-process bioburden reduction step that meets the USP <71> Standard for Sterility, thus, maintaining the integrity of the tissue biologically and biomechanically and eliminating the need for Terminal Sterilization like other processors use. MTF does not employ the use of harsh chemicals and terminal radiation in the processing of AmnioBand®, as these methods can have damaging effects on bone and soft tissue, as well as on the delicate nature of the acellular dermal matrix and placental tissues. MTF has performed all testing and safety. Before the placenta is donated, MTF conducts donor screening to ensure the donation of tissues is in accordance with current policies and procedures approved by the MTF Medical Board of Trustees. Donor blood samples taken at the time of recovery were tested for HBV, HCV, HIV-1/2, and syphilis by a facility that is CLIA certified and registered with the FDA.

### Swine Model and Surgery: Swine in the experimental groups were randomized, with MI only and MI+HPAC surgeries assigned alternative schedules for assurance of experimental reproducibility and consistency. Animals were acclimated in the University Animal Care (UAC) facility for 72 hours at a minimum. Following a 12-hour pre-operative fast, swine in MI only (n=4) and MI+HPAC (n=3) group were sedated and anesthetized prior to undergoing a percutaneous occlusion distal to the second diagonal of the LAD for 45 minutes.

Following the MI, swine in the MI+HPAC group underwent a median hemi-sternotomy. The LV was exposed, and the HPA membrane (AmnioBand® Matrix, MTF Biologics, Edison, NJ) (5cmx6cm) was sutured to the infarcted zone using 6-0 prolene. After treatment, pericardial closure was secured with pericardial membrane (Gore Preclude, Gore-Tex, Flagstaff, AZ, Ref: 1PCM103, Lot: 7612359), and incision was closed in a standard fashion.

### Swine Echocardiography: Using a 4-11-MHz transducer (GE LogiQ E, 8C-RS probe; GE Medical Systems, USA), standard two-dimensional parasternal long- and short-axis views at different levels (basal, mid, apical) were imaged. Data were analyzed offline using the integrated LogiQ E analysis features (Rev. 4). The data were obtained in triplicate and averaged.

### Determination of Swine Infarct Size and Histological Staining: Upon extraction of the LV cores, the explanted heart was semi-frozen at -20^º^C and sectioned from apex to base into 0.5cm thick short-axis segments using a meat slicer. The slices were weighed and then stained with 1% 2,3,4-triphenyl tetrazolium chloride (TTC, Sigma-Aldrich) solution and incubated in a water bath at 37^º^C for 30 minutes. The infarcted myocardium stained white (area of necrosis, AON) and viable tissue with oxidative activity stained red. Slices were digitally photographed at 8X magnification. AON, LV, RV, and whole slices were measured for each section using image analysis software (Image J 1.52a), and infarction size was reported as a percentage of AON to LV (unpaired t-test). Six assessors were blinded and independently evaluated the slices.

FFPE tissue blocks were stained with hematoxylin and eosin (H&E) to visualize overall tissue morphology and cellular infiltration, picro-sirius red (PSR) to detect collagen fibers as a marker for scar development according to standard protocols. PSR was visualized using a 5X objective under Alexa 488 (488nm in green) and Texas red (586nm in red) using Zeiss universal microscope (Axio Imager M1, Zeiss, Oberkochen, Germany). Collagen and fibrosis appear red while live, highly-ordered sarcomeres appear green. Overlaid image of both wavelengths was digitized and then analyzed by ImageJ software (Image J 1.52a).

*Swine RNA Sample Preparation and Sequencing*: Following excision of LV core from IZ, BZ, and RZ, samples were rinsed in 1X PBS (10x PBS Solution, 1.37M NaCl, 0.027M KCl and 0.119M phosphates, pH 7.4, Fisher BioReagents, Ref: BP3994) to remove any residual blood. Tissue was submerged in RNAlater® (Sigma-Aldrich, St. Louis, MO, Ref: R0901-500ML) and placed in 4ºC for 48 hours prior to storing in -80ºC.

The epicardial surface of the LV core biopsy (1/3 of the tissue) was utilized for RNA extraction. Tissue samples were submerged in 400μL of Trizol reagent (ThermoFisher, Carlsbad, CA, Ref:15596026) and ground for 30 seconds in a 2mL flat centrifuge tube using an OMNI THQ digital rotor-stator (OMNI, Kennesaw, GA, Ref:12-500) at 15,000RPM and hard tissue OMNI TipTM (OMNI, Kennesaw, GA, Ref: 30750H). Each sample was ground using a fresh tip to prevent cross contamination. Tubes were gently rocked back and forth to mix contents and were left undisturbed at room temperature for 10 minutes. Thereafter, 100μL of chloroform (Millipore-Sigma, Milwaukee, WI, Ref:288306) was added, mixed, and centrifuged (Eppendorf 5424R, Hamburg, Germany) at 15,000RPM for 5 minutes. The supernatant was transferred to a new tube, to which lysis buffer from the Qiagen RNEasy Plus Mini kit (Qiagen, Hilden, Germany, Ref: 74134) containing BME was added to bring samples up to a volume of 650μL. The total volume of sample was then transferred to a gDNA eliminator column from the Qiagen RNEasy Plus Mini kit and centrifuged in the Eppendorf at 8,000RPM for 30 seconds. The flow-through from the centrifugation step was then mixed in a 1:1 volume ratio of 70% EtOH, resulting in a final concentration of 35% EtOH by volume of the total sample volume. The subsequent steps were followed according to kit manufacturer’s protocol.

RNA samples were assessed for quality with an Advanced Analytics Fragment Analyzer (High Sensitivity RNA Analysis Kit – Ref: DNF-471-0500) and quantity with a Qubit RNA quantification kit (Qubit® RNA HS Assay KitAssay Kit – Ref: Q32852). RNA Quality Number (RQN) of samples ranged from 5.2 to 9.1.

Further quantity and quality validation were done prior to sequencing by Novogene (Novogene Co. Ltd., Sacramento, CA). RNA degradation and contamination were monitored on 1% agarose gels, and the purity of samples was assessed utilizing the NanoPhotometer® spectrophotometer (IMPLEN, CA, USA).
RNA integrity and quantitation were assessed using the RNA Nano 6000 Assay Kit of the Bioanalyzer 2100 system (Agilent Technologies, CA, USA).

Given satisfactory quality and quantity, samples were used for library builds. A total amount of 1μg RNA per sample was used as input material for the RNA sample preparations. Libraries were constructed using NEBNext®UltraTMRNA Library Prep Kit for Illumina® (NEB, USA) according to manufacturer’s recommendations. Index codes were added to attribute sequences to each sample. Poly-T oligo-attached magnetic beads were used to purify mRNA from the total RNA sample, and fragmentation was achieved using divalent cations under elevated temperature in NEBNext First Strand Synthesis Reaction Buffer (5X). Subsequently, second strand cDNA synthesis was performed using DNA Polymerase I and RNase H. Remaining overhangs were converted into blunt ends via exonuclease/polymerase activities. To prepare for hybridization, NEBNext Adaptor with hairpin loop structure were ligated following 3’ end adenylation. The AMPure XP system (Beckman Coulter, Beverly, USA) was used to isolate cDNA fragments of approximately 150-200 base pairs long. PCR was performed using Phusion High-Fidelity DNA polymerase, Universal PCR primers and Index (X) Primer following addition of 3μl USER Enzyme (NEB, USA) with size-selected, adaptor-ligated cDNA at 37°C for 15 minutes followed by 5 minutes at 95°C. Lastly, PCR products were purified (AMPure XP system), and library quality was assessed on the Agilent Bioanalyzer 2100 system.

The clustering of the index-coded samples was generated on a cBot Cluster Generation System using PE Cluster Kit cBot-HS (Illumina) according to the manufacturer’s instructions. The sequencing run was performed on an Illumina platform, and 125 bp/150 bp paired-end reads were generated.

### Swine RNAseq Analysis: The raw fastq format reads were processed by removing reads containing adapter, reads containing poly-N, and low-quality reads prior to aligning to the Sus scrofa (ftp://ftp.ensembl.org/pub/release-82/fasta/sus_scrofa/dna) genome using hisat2 2.1.0 (**Figure 6 and S3**) and using STAR (**Figure 5**).(50) Read numbers mapped to each gene were quantified using FeatureCounts v1.5.0-p3; Fragments Per Kilobase of transcript sequence per Millions base pairs sequenced (FPKM) for each gene was calculated based on gene length and read count and is utilized to estimate gene expression levels. (51, 52)

Using the DESeq 2 R package (1.14.1), differential expression analysis between two conditions/groups was performed using a model based on the negative binomial distribution. The resulting p-values were adjusted using the Benjamini and Hochberg’s approach for controlling the False Discovery Rate (FDR). Genes with an adjusted p-value <0.05 found by DESeq2 were assigned as differentially expressed.

Pathway analyses were performed. Gene Ontology (GO) enrichment analysis of differentially expressed genes was executed by the clusterProfiler R package to correct gene length bias. GO terms with adjusted p-value <0.05 were considered significantly enriched by differential expressed genes. Similarly, the Kyoto Encyclopedia of Genes and Genomes (KEGG) database (http://www.genome.jp/kegg/) was utilized using the clusterProfiler R package to understand high-level functions and utilities of the biological system based on statistically-enriched differential expression genes. These analyses provided the list of enriched pathways for our comparisons.

### Swine Proteome Analysis: Proteome analysis was performed for IZ cores for all three groups and RZ cores for MI only and MI+HPAC groups. The one-third of the LV core with the epicardial section of the RZ and IZ samples was obtained and weighed. Samples were homogenized with Katsumi buffer using the polytron homogenizer (Polytron® PT 10-35 GT, Kinematica, Ref: 13-874-617) at 30,000RPM for 10 second-pulses until tissue fragments were not visible in the sterile test tube. After 10 minutes of incubation on ice, contents were centrifuged (Microcentrifuge Heraeus™ Fresco™, Thermo Fisher, Ref: 75002421) at 13,300RPM for 15 minutes at 4°C. The supernatant was removed, and protein concentrations were determined using the Bradford method.

In-gel digestion of the LV tissue was performed, resulting peptides were purified by C18-based desalting as previously described, and dried peptides were resuspended in 6μl of 0.1% FA (v/v) followed by sonication for two minutes. (53) Final sample of 2.5μl was then analyzed by mass spectrometry.

The Scaffold output file was created and then post-processed in Progenesis QI (version 2.4, Nonlinear Dynamics Ltd., Newcastle upon Tyne, UK) to perform ion-intensity based label-free quantification. Due to variation in row 1 and 2 gel slices, data from these rows were excluded for proteomic analysis. A peak list of fragment ion spectra from only the top eight most intense precursors of a feature was exported in Mascot generic file (.mgf) format and searched against the pig SwissProt_2018 TrEMBL database (40,708 entries) database using Mascot (Matrix Science, London, UK; version 2.4) using the default probability cut-off score.

Protein and peptide quantification and logarithmic transformation were performed. Extracted ion abundance was normalized in a run to those in a reference run. Statistical analysis of protein abundance was performed in transformed peak area data (sin^-1^(peak area)) using analysis of variance in Progenesis QI. For statistical analysis, significance (p<0.05) was determined using 1-way ANOVA (Progenesis QI) and t-tests with two-tailed distribution and unequal variance (Excel). Progenesis QI proteome file was uploaded to Perseus for proteomics comprehensive analysis and visualization. (54) Heatmap clustering matrices and significant (p<0.05) proteins and peptides were identified for GO and KEGG pathway enrichment using DAVID (version 6.8). (55)

Western Blotting: Protein concentration of LV lysates was quantified using the Bradford method. The lysates were then combined with SDS sample loading buffer (4% SDS, 0.0625M Tris-HCl, 10% glycerol, 0.02% bromphenol blue, 8.0M urea). The 30μg of homogenates were then separated by 12% SDS-PAGE, and the gels were either stained with Bio-Safe Coomassie G-250 Stain (1610406, BioRad, Hercules, CA) or transferred to a Polyvinylidene fluoride (PVDF) membrane for subsequent western blotting.

PVDF membranes of swine cardiac LV lysates were incubated in 50% LI-COR blocking buffer (927-50000, LI-COR, Lincoln, NE) at room temperature for 1 hour. The membranes were probed with commercially available primary antibodies as described in Table 2.6 at 4°C overnight. Following primary antibody (see **Table S1**) incubation and 1X PBST (10X PBS Solution, 1.37M NaCl, 0.027M KCl and 0.119M phosphates, 0.1% Tween-20, pH 7.4, Fisher BioReagents, Ref: BP3994) washes membranes were incubated with secondary antibodies at 1:15,000 dilution (1155035-003, Jackson ImmunoResearch Laboratories, West Grove, PA, Lot: 120344) at room temperature for 60 minutes. Membranes were imaged using an Odyssey CLx Infrared Imaging System (LI-COR, Lincoln, NE). All protein band optical densities were analyzed in LI-COR Image Studio Lite (software version 5.1), and according to accepted guidelines, untreated control LV samples were loaded into each gel for multi-blot comparisons. Protein blots were normalized to loading control proteins, beta-tubulin, after adjusting for total protein quantified from PVDF membranes stained with Ponceau S.

| **Table S1: Antibody source and dilution** | | | |
| --- | --- | --- | --- |
| **1⁰ Ab** | **Cat. No.** | **Vendor** | **Dilution** |
| HSP90 | Ab13492 | Abcam | 1:1000 |
| HSP70 | Ab5439 | Abcam | 1:1000 |
| HSP40 | Ab69402 | Abcam | 1:1000 |
| β-tubulin | 9249P | Thermo Scientific | 1:2000 |

### Murine Model and Surgery: A total of 13 male C57Bl/6J adult mice (Harlan, 8-10 weeks old) were randomly assigned to the MI only (n=6) group or MI+HPA (n=7) (Amnio Technology, LLC) group. The mice were housed together (2-4 per container) based on their designated experimental group in a temperature-controlled housing contained and maintained on a 12-hr light-dark cycle. Every mouse had access to food and water ad libitum throughout the duration of the experiments.

All mice prior to surgery were weighed and anesthetized. For the chronic heart failure (CHF) mice, MI was induced by ligation of the left coronary artery (LCA) as was previously described.(56) Under anesthesia (2.5% isoflurane in a mixture of air and O2), a thoracotomy was performed at the fourth left intercostal space and the LCA permanently ligated to induce a MI. Occlusion of the LCA was confirmed by observing blanching, a slight change in color of the anterior wall of the left ventricle downstream of the ligature. The HPA xenograft was placed onto the area of occlusion sutured in place such that approximately one-half to two-thirds of the infarcted, blanched area was covered. Echocardiography was performed at baseline and 10- and 28-days post-MI to assess ventricular function, cardiac morphometry, and remodeling.

*Murine Echocardiography:* Transthoracic echocardiography was performed prior to MI surgery and again at 10- and 28-days post-MI using a Visual Sonics Vevo 2100 high-resolution imaging system (Visual Sonics, Toronto, ON, Canada) and a 25-MHz transducer. The chests of animals were shaved with a chemical hair remover. Anesthesia was maintained by 1% isoflurane balanced with oxygen. Body temperature was maintained using a heated platform. Respiratory rates and electrocardiograms were monitored throughout the study. The echocardiographic procedure was performed in conscious mice to study cardiac function at more physiological heart rates and to eliminate any anesthesia effects.(57)

Two-dimensional M-mode echocardiographic images were obtained from the parasternal short-axis views at the level of the mid-ventricles. Cardiac chamber dimensions and the left ventricular wall thickness were measured. Ejection fraction (EF), left ventricular volume (LV Vol), left ventricular posterior wall thickness (LVPW) and internal dimension (LVID) were measured from the M-mode images. Data was analyzed offline using Vevo 2100 analytic software. The data were obtained in triplicate and averaged.

## SUPPLEMENTAL DATA

### IRI Demonstrates Different Spatial Adaptations in Swine Infarct and Remote Zones: Local effects following IRI are pertinent to understanding the central and peripheral effects of injury. A comparison of proteomic results between IZ and RZ within the MI only group identifies 11 upregulated and 21 downregulated proteins (**Supplemental** **Figure S4A**). Following functional annotation using GO and KEGG, a manual search of the significant proteins was performed. Identified proteins are associated with decreased energy reserves (TIGAR, PIK3R1), decreased cell proliferation (KIAA0586, PRKACB), changes in transcription (SBNO2, MYCT1, FUS, PPP1R13L) and translation (GON7) regulation, downregulation of mitochondrial proteins (RARS2, CCDC51, BID), and decreased apoptosis and autophagy (ATG7, BID, PPP1R13L, TAB1, HSP90AB1, TBC1D25, TIGAR). Moreover, changes in immune processes such as upregulation of PPP6C suggest regulation in the differentiation of regulatory T cells. However, decreases in TMEM9B and SBNO2 imply regulation of pro-inflammatory cascades; that is, TMEM9B enhances the production of pro-inflammatory cytokines, and SBNO2 additively represses NF-ĸB activation. A decrease in P2RX7 is also observed and signifies reduced ATP-dependent lysis of macrophages. Another noteworthy finding is the upregulation of FLG2 in MI only IZ, which is known to mediate the formation of fibrin-rich microthrombi in cardiac microvascular endothelial cells following IRI.

### HPAC Modulates Intracellular Transport Proteins and Alters Energy Dynamics in Swine Model: A similar comparison was performed with MI+HPAC IZ to MI+HPAC RZ. Volcano plot illustrates 27 and 157 upregulated and downregulated genes, respectively (**Supplemental** **Figure S4B**). GO and KEGG functional annotation disclosed response to ischemia, apoptosis, energy metabolism, and calcium signaling processes. Further interrogation of the proteins revealed changes to protein trafficking, autophagy, and angiogenesis. Taken together, we believe the short- and long-term response to HPAC should be investigated to characterize mechanisms and chronic remodeling, respectively.

### Network Analysis Encouraged Further Interrogation

To determine the correlation between the transcriptome and proteome profiles of MI only and MI+HPA groups, we performed a network analysis to study the relationship of the RNAseq and proteomic data. Their respective fold changes were mapped with genes on the x-axis and proteins on the y-axis. To do so, we matched genes and proteins based on their UniProtID and Ensembl ID. Direct comparisons of each zone were compared to the groups. No noteworthy overlapping gene-protein IDs were identified. This was rather anticipated given that functional annotation of swine genes and proteins from current databases is currently incomplete, thereby limiting biological interpretation. Pipeline tools for data integration from high throughput RNA sequencing, proteomics, and metabolomics allow matching individual ‘omic data matrices and for easier experimentation and reproducibility. Our goal for future studies with larger sample size is to utilize these tools to build a model that better elucidates the phenotypes of the three groups.

### Response to HPA Application in the Mouse Infarct Model: We first examined the impact of HPA xenografts on cardiac remodeling using a murine model of MI. Following permanent ligation of the left coronary artery to instigate MI in 13 mice, 7 mice received an HPA patch sutured to the epicardial surface of the infarcted area while the remaining 6 mice served as MI controls. Serial echocardiography was performed at baseline (prior to MI surgery) and at 10- and 28-days post-MI to monitor progression of cardiac dysfunction as the infarcted myocardium remodeled. By 10 days following MI, left-ventricular internal dimension during systole (LVIDs) was significantly increased in both MI and MI+HPA hearts over baseline LVIDs (**Figure S5A**). This elevation in LVIDs persisted through the 28-day study period. MI and MI+HPA mice showed worsening morphology with an increase in left ventricular volume during systole (LV Vol s; **Figure S5B**) and a decrease in left ventricular posterior wall during systole (LVPWs; **Figure S5C**) at 10- and 28-days post-MI. This is indicative of ventricular dilation and a worsening of cardiac function. The resultant impact of these morphological changes in ventricular chamber dimensions following MI was a decline in cardiac function (ejection fraction; EF) by 10 days (**Figure S5D**) that was significantly different from baseline in both experimental groups. Again, this decrease in EF did not worsen at 28 days post-MI.

At 28 days post-MI, hearts were excised, fixed and stained with H&E. Representative sections (5x magnification) of H&E-stained hearts are illustrated in **Figure S6** for MI and MI+HPA whole hearts in the long-axis view. The HPA showed efficient and complete engraftment to the native myocardium. There was substantial loss of myocardium within the infarcted segment in mice subjected to MI at 10- and 28-days (**Figure S6A, left panel**) or MI+HPA (**Figure S6A, right panel**). However, wall thickness was preserved in the region of the infarcted area covered by HPA (**Figure S6A, right panel**). At 20x magnification, the increase in wall thickness due to cellular accumulation is evident in the MI+HPA myocardium (**Figure S6B, right panel**) when compared to myocardium without the patch (**Figure S6B, left panel**). Compared to non-infarcted myocardium, the wall thickness of infarcted myocardium not covered by HPA was approximately one-tenth the wall thickness. Although significantly less than non-infarcted myocardium, myocardium that was covered by HPA had, on average, a significant 3-fold greater wall thickness over the wall thickness of the infarcted myocardium not covered by HPA **(Figure S6C)**.

**SUPPLEMENTAL FIGURE LEGENDS**

**Figure S1**: **Schematic Diagram of the Swine Study**. Timeline illustrates swine randomized to MI only (n=4) or MI+HPAC (n=3) groups underwent 45-minute ischemia reperfusion injury with or without HPAC membrane therapy with scheduled blood collection and echocardiography. Four swine remained as controls. On post-operative day (POD) 14, swine were sacrificed for tissue harvest for histological, transcriptomic, and proteomic analyses. On the right, a representation of sutured HPAC membrane placement onto infarcted LV apex is presented.

**Figure S2:** **Excised MI only and MI+HPAC Swine Hearts**. **(A)** MI only swine had a virgin chest without adhesions. Infarcted area could be visualized in the apex. **(B)** A punch biopsy was used to core the infarct, border, and remote zones. **(C)** Intact sutured human placental amniotic (HPAC) membrane can be grossly visualized and demonstrates partial engraftment. **(D)** MI+HPAC swine previously underwent a median hemi-sternotomy and was noted to have chest wall adhesions. Panel D illustrates the most severe case. **(E-F)** The white arrows point to the sutured HPAC membrane to the LV apex.

**Figure S3**: **Transcriptome Profile of Remote Zone (RZ) of Swine MI+HPAC to Control Group**. **(A)** Volcano plot with 635 significantly expressed genes presented as -log_10_(p-adj) and log_2_(fold change) of RZ of MI+HPAC to control group (364 upregulated [red], 271 downregulated [green]). **(B)** C_net_ plot of GO biological processes with significantly expressed genes. **(C)** Dot plot of GO analysis of significantly differentially expressed genes. **(D)** GO analysis of top 20 pathways of the differentially expressed genes. **(E)** Table of significantly expressed gene related to GO wound healing pathway (GO:0042060) with upregulation [red] and downregulation [green] defined. N=3 for each group. Significant criteria of differential expression genes are -log_10_(p-adj) > 1.3 and |log_2_(FoldChange)| > 1.

**Figure S4**: **Proteomic Analysis of Infarct to Remote Zone of MI Only and MI+HPAC Swine Groups**. **(A)** Volcano plot of MI only infarct zone (IZ) to remote zone (RZ) show 11 upregulated [pink] and 21 downregulated [teal] proteins as -log_10_(p-value) and log_2_(fold change). **(B)** Volcano plot of MI+HPAC IZ to RZ show 27 upregulated and 157 downregulated proteins as -log_10_(p-value) and log_2_(fold change). N=3 per group. P<0.05.

**Figure S5. Echocardiographic Parameters of Ventricular Function and Morphometry in Mouse MI and MI+HPA Hearts**. **(A)** LVIDs is LV internal diameter at end-systole. **(B)** LV Vol s is LV volume at end-systole. **(C)** LVPWs is LV posterior wall thickness at end-systole. **(D)** Ejection fraction (EF%). Data presented as Mean ± S.E.M. Experimental group numbers are as follows, MI, n=6; MI+HPA, n=7; p<0.05 from baseline.

**Figure S6. Cardiac Histology and Morphometry in MI Only and MI+HPA Mice. (A)** Representative images of H&E stained hearts in longitudinal sections of MI and MI+HPA mice. **(B)** Representative H&E stained hearts at 20x magnification of infarcted myocardium (**left panel**) and infarcted myocardium covered by membrane (**right panel**). **(C)** From the histological images, wall thickness was measured in the each of the hearts that received MI and membrane at three regions non-infarcted myocardium, infarcted myocardium and infarcted myocardium covered by HPA. Three measurements in each region were averaged and then normalized to non-infarcted region for each heart. Then, each region was averaged among all hearts and normalized to non-infarcted region. Data presented as mean ± S.E.M. N=7 for each region; *p<0.05 from non-infarcted myocardium; # p<0.05 from infarcted myocardium.
